# Supplementary material for: Determinants of non-communicable disease management among support staff in Putrajaya, Malaysia: The mediating role of attitude
Source: PLoS One. 2026 Apr 30;21(4):e0347528. doi: 10.1371/journal.pone.0347528 (PMC13132246; doi:10.1371/journal.pone.0347528)
Supplement: S2 File — (DOCX) [file pone.0347528.s002.docx]

**KNOWLEDGE OF NCD**

| 1. Item | No | Not Sure | Yes |
| --- | --- | --- | --- |
| I know about non-communicable diseases. | 1 | 2 | 3 |

| 1. Items | No | Not Sure | Yes |
| --- | --- | --- | --- |
| 1. I know that diabetes is a non-communicable disease. | 1 | 2 | 3 |
| 1. I know that tuberculosis is a non-communicable disease. | 1 | 2 | 3 |
| 1. I know that asthma is a non-communicable disease. | 1 | 2 | 3 |
| 1. I know that dengue fever is a non-communicable disease. | 1 | 2 | 3 |
| 1. I know that heart disease is a non-communicable disease. | 1 | 2 | 3 |
| 1. I know that leptospirosis is a non-communicable disease. | 1 | 2 | 3 |

| 1. Items | No | Not Sure | Yes |
| --- | --- | --- | --- |
| 1. I know that regular consumption of foods high in fat (for example nasi lemak and fried chicken) is a risk factor for heart attacks. | 1 | 2 | 3 |
| 1. I know that eating a balanced diet is one of the best practices to avoid the risk of a heart attack. | 1 | 2 | 3 |
| 1. I know that smoking is a risk factor for a heart attack. | 1 | 2 | 3 |
| 1. I know that doing physical activity is one of the best practices to avoid the risk of heart attack. | 1 | 2 | 3 |

| 4**.** Items | No | Not Sure | Yes |
| --- | --- | --- | --- |
| 1. I know that high blood pressure is a risk factor for stroke. | 1 | 2 | 3 |
| 1. I know that doing physical activity regularly is one of the best practices to avoid the risk of stroke. | 1 | 2 | 3 |
| 1. I know that having a balanced diet is one of the best practices to avoid the risk of stroke. | 1 | 2 | 3 |
| 1. I know that obesity is a risk factor for stroke. | 1 | 2 | 3 |

| 1. Items | No | Not Sure | Yes |
| --- | --- | --- | --- |
| 1. I know that individuals with a systolic reading above 140 have high blood pressure. | 1 | 2 | 3 |
| 1. I know that people with high blood pressure patients need to take medication as prescribed. | 1 | 2 | 3 |
| 1. I know that individuals with obesity have a high risk of getting high blood pressure disease. | 1 | 2 | 3 |
| 1. I know that a person with high blood pressure needs to undergo regular follow-up treatment at a health center. | 1 | 2 | 3 |

| 1. Items | No | Not Sure | Yes |
| --- | --- | --- | --- |
| 1. I know that higher carbohydrate intake from starchy foods such as rice, potatoes and bread is a risk of diabetes. | 1 | 2 | 3 |
| 1. I know that someone who has a family member with diabetes is at risk of getting diabetes. | 1 | 2 | 3 |
| 1. I know that people with type 2 diabetics need to take non-insulin injectable drugs. | 1 | 2 | 3 |
| 1. I know that diabetes can cause kidney failure. | 1 | 2 | 3 |

| 1. Items | No | Not Sure | Yes |
| --- | --- | --- | --- |
| 1. I know that shortness of breath is a symptom of chronic obstructive pulmonary disease (COPD). | 1 | 2 | 3 |
| 1. I know that increased phlegm is a sign of chronic obstructive pulmonary disease (COPD). | 1 | 2 | 3 |
| 1. I know that breathing sounds followed by wheezing and noisy breathing is chronic obstructive pulmonary disease (COPD). | 1 | 2 | 3 |
| 1. I know that a chronic coughing for more than three months is a sign of chronic obstructive pulmonary disease (COPD). | 1 | 2 | 3 |

**ATTITUDE TOWARDS NCD**

| 1. At my current age, I feel that it is more important to take care of my health. | 1 | 2 | 3 | 4 | 5 |
| --- | --- | --- | --- | --- | --- |
| 1. I feel it is important to practice a balanced diet continuously. | 1 | 2 | 3 | 4 | 5 |
| 1. I’m trying to control non-communicable diseases from getting worse. | 1 | 2 | 3 | 4 | 5 |
| 1. At my current age, regular physical activity is important to me in order to maintain health. | 1 | 2 | 3 | 4 | 5 |
| 1. I feel it is necessary to bring medicine with me when visiting relatives who are far away for a week. | 1 | 2 | 3 | 4 | 5 |
| 1. I feel it is necessary to consume food according to the calories I need for my daily activities. | 1 | 2 | 3 | 4 | 5 |
| 1. In my opinion, taking the medicine given by the doctor according to the prescribed schedule can control the disease. | 1 | 2 | 3 | 4 | 5 |
| 1. In choosing food, I will make sure to eat foods that contain less sugar. | 1 | 2 | 3 | 4 | 5 |
| 1. If my family members smoke, I will try to advise them to stop smoking. | 1 | 2 | 3 | 4 | 5 |
| 1. In choosing food, I will make sure to eat food that contains less fat. | 1 | 2 | 3 | 4 | 5 |
| 1. I would not continue to consume excessive salt. | 1 | 2 | 3 | 4 | 5 |
| 1. In my opinion, a person suffering from high blood pressure should undergo a blood pressure check during follow-up treatment. | 1 | 2 | 3 | 4 | 5 |
| 1. I will provide financial assistance if my family member has a stroke | 1 | 2 | 3 | 4 | 5 |
| 1. I would be willing to take care of my family members if they had a stroke. | 1 | 2 | 3 | 4 | 5 |
| 1. I will encourage female family members to undergo breast cancer screening at the health center. | 1 | 2 | 3 | 4 | 5 |

**INTERPERSONAL FACTORS**

**Over the past 3 months**

| **Family** |  |  |  |  |  |
| --- | --- | --- | --- | --- | --- |
| 1. I do exercise with my family. | 1 | 2 | 3 | 4 | 5 |
| 1. My family listened carefully about my health or illness. | 1 | 2 | 3 | 4 | 5 |
| 1. My family encouraged me to do the things I needed to do for my health. | 1 | 2 | 3 | 4 | 5 |
| 1. My family selects or requests healthy food choices when we eat together. | 1 | 2 | 3 | 4 | 5 |
| 1. I share healthy low-fat recipes with family members | 1 | 2 | 3 | 4 | 5 |
| 1. My family reminds me to take medicine. | 1 | 2 | 3 | 4 | 5 |
| 1. My family bought or prepared healthy food for me | 1 | 2 | 3 | 4 | 5 |
| 1. Family support is important to me in managing non-communicable diseases. | 1 | 2 | 3 | 4 | 5 |

**Over the past 3 months**

| **Friends** |  |  |  |  |  |
| --- | --- | --- | --- | --- | --- |
| 1. I do exercise with friends. | 1 | 2 | 3 | 4 | 5 |
| 1. My friends listened carefully about my health or illness. | 1 | 2 | 3 | 4 | 5 |
| 1. My friends encouraged me to do the things I needed to do for my health. | 1 | 2 | 3 | 4 | 5 |
| 1. My friends selects or requests healthy food choices when we eat together. | 1 | 2 | 3 | 4 | 5 |
| 1. I share healthy low-fat recipes with family friends. | 1 | 2 | 3 | 4 | 5 |
| 1. My friends remind me to take medicine. | 1 | 2 | 3 | 4 | 5 |
| 1. My friends bought or prepared healthy food for me | 1 | 2 | 3 | 4 | 5 |
| 1. Friends are important to me in managing non-communicable diseases. | 1 | 2 | 3 | 4 | 5 |

**ORGANIZATIONAL FACTOR**

**Over the past 3 months**

| 1. I have a flexible work schedule that can be adjusted to meet my needs. | 1 | 2 | 3 | 4 | 5 |
| --- | --- | --- | --- | --- | --- |
| 1. My workplace offered wellness programs or fitness facilities. | 1 | 2 | 3 | 4 | 5 |
| 1. My workplace provides healthy food. | 1 | 2 | 3 | 4 | 5 |
| 1. My workplace had rules or policies that made it easier for me to manage my health or illness (such as no smoking rules or time off work to exercise). | 1 | 2 | 3 | 4 | 5 |
| 1. Coworkers take over my job when I feel unwell or to manage my illness. | 1 | 2 | 3 | 4 | 5 |
| 1. I had control over my job in terms of making decisions and setting priorities. | 1 | 2 | 3 | 4 | 5 |
| 1. My employer provides time off to do health care or fitness activities. | 1 | 2 | 3 | 4 | 5 |
| 1. Workplace support is important to me in managing non-communicable diseases. | 1 | 2 | 3 | 4 | 5 |

**COMMUNITY FACTORS**

**Over the past 3 months**

| **Community** |  |  |  |  |  |
| --- | --- | --- | --- | --- | --- |
| 1. The pharmacy I went to provides good information about non-communicable diseases. | 1 | 2 | 3 | 4 | 5 |
| 1. I noticed that the stores I frequently shop have healthy low-fat foods. | 1 | 2 | 3 | 4 | 5 |
| 1. My community made an effort to include groups and organizations of people with chronic diseases in community activities. | 1 | 2 | 3 | 4 | 5 |
| 1. I found that people in my community accepted those who have non-communicable diseases. | 1 | 2 | 3 | 4 | 5 |
| 1. I ate at a restaurant that offered a variety of tasty, low-fat food   choices. | 1 | 2 | 3 | 4 | 5 |
| 1. I used public transportation to get somewhere I was going. |  |  |  |  |  |
| 1. I went to parks for picnics, walks, or other outings. | 1 | 2 | 3 | 4 | 5 |
| 1. The local community environment is important to me in managing non-communicable diseases. | 1 | 2 | 3 | 4 | 5 |

**Over the past 3 months**

| **Neighborhood** |  |  |  |  |  |
| --- | --- | --- | --- | --- | --- |
| 1. I walked or exercised outdoors in my neighborhood. | 1 | 2 | 3 | 4 | 5 |
| 1. I talked to neighbors or others who have experience living with a non-communicable diseases. | 1 | 2 | 3 | 4 | 5 |
| 1. My neighbors and I gotten together for activities such as barbecues, parties or holiday parties. | 1 | 2 | 3 | 4 | 5 |
| 1. The grocery stores where I shopped had a good supply of fresh fruits and vegetables. | 1 | 2 | 3 | 4 | 5 |
| 1. I walked or did other exercise activities with neighbors. | 1 | 2 | 3 | 4 | 5 |
| 1. I shared recipes or discussed healthy eating ideas with neighbors. |  |  |  |  |  |
| 1. Neighborhood factors are important to me in managing non-communicable diseases. | 1 | 2 | 3 | 4 | 5 |

**Over the past 3 months**

| **Health services** |  |  |  |  |  |
| --- | --- | --- | --- | --- | --- |
| 1. The doctor or other health advisor (nurse, dietician) clearly explained what I needed to do to manage non-communicable diseases (If you have not had any doctor visits in the past 3 months, think back to the last visit you had.) | 1 | 2 | 3 | 4 | 5 |
| 1. Doctors or other health advisors provide support during appointments. | 1 | 2 | 3 | 4 | 5 |
| 1. The doctor involved me as an equal partner in making decisions about non-communicable disease management strategies and goals. | 1 | 2 | 3 | 4 | 5 |
| 1. The doctor or other health care advisor listened carefully to what I had to say about my health or illness. | 1 | 2 | 3 | 4 | 5 |
| 1. The doctor or other health advisor (nurse, dietician) answered my questions and addressed my concerns during the appointment. | 1 | 2 | 3 | 4 | 5 |
| 1. The doctor or health care provider (medical lab technologist, pharmacist) provider thoroughly explained the results of tests I had done (e.g., cholesterol, blood pressure, or other laboratory tests). | 1 | 2 | 3 | 4 | 5 |
| 1. Healthcare team (doctors, nurses, nutritionists, medical laboratory technologists, and pharmacists) are important to me in managing non-communicable diseases. | 1 | 2 | 3 | 4 | 5 |

**Over the past 3 months**

| **Organization Community** |  |  |  |  |  |
| --- | --- | --- | --- | --- | --- |
| 1. I called a national or local health organization for information about non-communicable diseases. | 1 | 2 | 3 | 4 | 5 |
| 1. People at my work, religious groups, or other organizations to which I belong showed understanding and support for management efforts of non-communicable diseases. | 1 | 2 | 3 | 4 | 5 |
| 1. People at my work, religious groups, or other organizations have shown understanding and support in managing non-communicable diseases. | 1 | 2 | 3 | 4 | 5 |
| 1. I participated in walks or other activities for health organizations | 1 | 2 | 3 | 4 | 5 |
| 1. I attended free or low-cost activities (such as weight loss programs, religious gatherings, and hospital programs) that support non-communicable disease management. | 1 | 2 | 3 | 4 | 5 |
| 1. I volunteered my time for local organizations of non-communicable disease management. | 1 | 2 | 3 | 4 | 5 |
| 1. I attended wellness programs or used fitness facilities. |  |  |  |  |  |
| 1. I called or visited a local health organization or hospital to find information, view a video, or check out written materials. | 1 | 2 | 3 | 4 | 5 |
| 1. I used community resources to help manage non-communicable diseases such as senior centers, community centers, or programs at shopping malls. | 1 | 2 | 3 | 4 | 5 |
| 1. Community organizations are important to me in managing non-communicable diseases. | 1 | 2 | 3 | 4 | 5 |

**SOCIETAL FACTORS**

**Over the past 3 months**

| **Health Policy** |  |  |  |  |  |
| --- | --- | --- | --- | --- | --- |
| 1. Health insurance can help to cover medical treatment and healthcare services. | 1 | 2 | 3 | 4 | 5 |
| 1. Health insurance covers most of the costs of my medical   needs including medicine and treatment. | 1 | 2 | 3 | 4 | 5 |
| 1. Health policy resources are important to me in managing non-communicable diseases. | 1 | 2 | 3 | 4 | 5 |

**Over the past 3 months**

| **Mass Media** |  |  |  |  |  |
| --- | --- | --- | --- | --- | --- |
| 1. I read articles in newspapers or magazines about people who have successfully managed non-communicable diseases. | 1 | 2 | 3 | 4 | 5 |
| 1. I saw billboards or other advertisements that encouraged not smoking, low-fat eating, or regular exercise. | 1 | 2 | 3 | 4 | 5 |
| 1. I watch television or listen to radio programs that focus on health or   lifestyle issues | 1 | 2 | 3 | 4 | 5 |
| 1. I heard things on the news that encouraged me to take good care of   my health. | 1 | 2 | 3 | 4 | 5 |
| 1. I used the Internet or World Wide Web to find or share information   about on-communicable diseases. | 1 | 2 | 3 | 4 | 5 |
| 1. I watch TV programs or listen to radio programs that realistically portray what it’s like living with non-communicable diseases. | 1 | 2 | 3 | 4 | 5 |
| 1. I watch TV programs or listen to radio programs that provide good information on non-communicable diseases. | 1 | 2 | 3 | 4 | 5 |
| 1. Mass media is important to me in managing illness or non-communicable diseases. | 1 | 2 | 3 | 4 | 5 |

**MANAGEMENT OF NON-COMMUNICABLE DISEASES**

**Over the past 3 months**

| 1. I have taken time for myself by doing non-communicable disease management practice activities . | 1 | 2 | 3 | 4 | 5 |
| --- | --- | --- | --- | --- | --- |
| 1. I reward myself when I succeed in following non-communicable disease management practices. | 1 | 2 | 3 | 4 | 5 |
| 1. I focus on the things I do well in non-communicable disease management practice. | 1 | 2 | 3 | 4 | 5 |
| 1. I seek insights to help me in my non-communicable disease management practice. | 1 | 2 | 3 | 4 | 5 |
| 1. I evaluate or review achievements in the following non-communicable disease management practices. | 1 | 2 | 3 | 4 | 5 |
| 1. I pray/worship/meditate to help me in following non-communicable disease management practices. | 1 | 2 | 3 | 4 | 5 |
| 1. I organize my daily schedule so that it is easy to do activities that help with non-communicable disease management. | 1 | 2 | 3 | 4 | 5 |
| 1. Non-communicable disease management is important to me for long-term health. | 1 | 2 | 3 | 4 | 5 |
