# Supplementary material for: Determinants of non-communicable disease management among support staff in Putrajaya, Malaysia: The mediating role of attitude
Source: PLoS One. 2026 Apr 30;21(4):e0347528. doi: 10.1371/journal.pone.0347528 (PMC13132246; doi:10.1371/journal.pone.0347528)
Supplement: Attachment — Submitted filename: PONE-D-25-10747_reviewer.pdf [file pone.0347528.s003.pdf]

PLOS ONE

Mediating Role of Attitude on the Relationship Between Socio-Ecological Factors and Non-Communicable Disease Management among Support Staff in Putrajaya, Malaysia

--Manuscript Draft--

**Manuscript Number:**	PONE-D-25-10747
**Article Type:**	Research Article
**Full Title:**	Mediating Role of Attitude on the Relationship Between Socio-Ecological Factors and Non-Communicable Disease Management among Support Staff in Putrajaya, Malaysia
**Short Title:**	Mediating Role of Attitude on the Relationship Between Socio-Ecological Factors and NCD Management
**Corresponding Author:**	Haliza Abdul Rahman, PhD<br>Universiti Putra Malaysia<br>Serdang, Selangor MALAYSIA
**Keywords:**	Attitude; Socio-ecological factors; Non-communicable Disease Management; Support staff; Mediation Effects; Malaysia
**Abstract:**	Non-communicable disease (NCD) is a major global health issue that contributes to morbidity and mortality problems across countries including Malaysia. Low-income groups are one of the most vulnerable and at high-risk groups for NCD. However, no empirical study has dealt with the mediating effect of attitude between socio-ecological factors (intrapersonal, interpersonal, organizational, community and societal factors) and NCD management. Thus, this study aimed to examine the mediating role of attitude on the relationship between socio-ecological factors and NCD management. This study employs a cross-sectional survey of 453 support staff at selected ministries in Putrajaya, Malaysia. Data collected included socio-demographic information, knowledge of NCD, attitude towards preventing NCD, and Chronic Illness Resources Survey (CIRS). Descriptive and Structural equation modeling (SEM) analysis were used to analyze data. The path analysis showed that attitude, community and societal factors effect on NCD management. Mediation effects showed a significant indirect effect of intrapersonal, community and societal factors on NCD management through attitude. These findings indicated that intrapersonal, community and societal factors are mediated by attitude, which impacts NCD management. This study suggests that the relevant stakeholders such as government, policymakers, health professionals and program managers pay attention to intrapersonal, community and societal factors to improve NCD management and enhance strategies, policies, and planning on NCD management among low-income groups in Malaysia.
**Order of Authors:**	Haliza Abdul Rahman, PhD
	Azrin Shah Abu Bakar
	Ahmad Iqmer Nashriq Mohd Nazan, PhD
**Additional Information:**	
**Question**	**Response**
**Financial Disclosure**<br><br>Enter a financial disclosure statement that describes the sources of funding for the work included in this submission. Review the submission guidelines for detailed requirements. View published research articles from *PLOS ONE* for specific examples.	The author(s) received no specific funding for this work.

Powered by Editorial Manager® and ProduXion Manager® from Aries Systems Corporation

This statement is required for submission and **will appear in the published article** if the submission is accepted. Please make sure it is accurate.<br><br>**Funded studies**<br>Enter a statement with the following details:<br>• Initials of the authors who received each award<br>• Grant numbers awarded to each author<br>• The full name of each funder<br>• URL of each funder website<br>• Did the sponsors or funders play any role in the study design, data collection and analysis, decision to publish, or preparation of the manuscript?<br><br>Did you receive funding for this work?	
**Competing Interests**<br><br>Use the instructions below to enter a competing interest statement for this submission. On behalf of all authors, disclose any competing interests that could be perceived to bias this work—acknowledging all financial support and any other relevant financial or non-financial competing interests.<br><br>This statement is required for submission and **will appear in the published article** if the submission is accepted. Please make sure it is accurate and that any funding sources listed in your Funding Information later in the submission form are also declared in your Financial Disclosure statement.<br><br>View published research articles from *PLOS ONE* for specific examples.	The authors have declared that no competing interests exist.

**NO authors have competing interests**<br><br>Enter: *The authors have declared that no competing interests exist.*<br><br>**Authors with competing interests**<br><br>Enter competing interest details beginning with this statement:<br><br>*I have read the journal's policy and the authors of this manuscript have the following competing interests: [insert competing interests here]*<br><br>* typeset	
**Ethics Statement**<br><br>Enter an ethics statement for this submission. This statement is required if the study involved:<br><br>• Human participants<br>• Human specimens or tissue<br>• Vertebrate animals or cephalopods<br>• Vertebrate embryos or tissues<br>• Field research<br><br>Write "N/A" if the submission does not require an ethics statement.<br><br>General guidance is provided below. Consult the submission guidelines for detailed instructions. **Make sure that all information entered here is included in the Methods section of the manuscript.**	Ethical approval to conduct this study was obtained from Ethics Committee for Research Involving Human Subject, Universiti Putra Malaysia with the reference number of JKEUPM-2024-301.

Powered by Editorial Manager® and ProduXion Manager® from Aries Systems Corporation

**Format for specific study types**<br><br>**Human Subject Research (involving human participants and/or tissue)**<br>• Give the name of the institutional review board or ethics committee that approved the study<br>• Include the approval number and/or a statement indicating approval of this research<br>• Indicate the form of consent obtained (written/oral) or the reason that consent was not obtained (e.g. the data were analyzed anonymously)<br><br>**Animal Research (involving vertebrate animals, embryos or tissues)**<br>• Provide the name of the Institutional Animal Care and Use Committee (IACUC) or other relevant ethics board that reviewed the study protocol, and indicate whether they approved this research or granted a formal waiver of ethical approval<br>• Include an approval number if one was obtained<br>• If the study involved *non-human primates*, add *additional details* about animal welfare and steps taken to ameliorate suffering<br>• If anesthesia, euthanasia, or any kind of animal sacrifice is part of the study, include briefly which substances and/or methods were applied<br><br>**Field Research**<br><br>Include the following details if this study involves the collection of plant, animal, or other materials from a natural setting:<br>• Field permit number<br>• Name of the institution or relevant body that granted permission	
**Data Availability**<br><br>Authors are required to make all data underlying the findings described fully available, without restriction, and from the time of publication. PLOS allows rare exceptions to address legal and ethical concerns. See the PLOS Data Policy and FAQ for detailed information.	Yes - all data are fully available without restriction

A Data Availability Statement describing where the data can be found is required at submission. Your answers to this question constitute the Data Availability Statement and **will be published in the article**, if accepted.<br><br>**Important:** Stating 'data available on request from the author' is not sufficient. If your data are only available upon request, select 'No' for the first question and explain your exceptional situation in the text box.<br><br>Do the authors confirm that all data underlying the findings described in their manuscript are fully available without restriction?	
**Describe where the data may be found in full sentences. If you are copying our sample text, replace any instances of XXX with the appropriate details.**<br><br>• If the data are **held or will be held in a public repository**, include URLs, accession numbers or DOIs. If this information will only be available after acceptance, indicate this by ticking the box below. For example: *All XXX files are available from the XXX database (accession number(s) XXX, XXX.*).<br>• If the data are all contained **within the manuscript and/or Supporting Information files**, enter the following: *All relevant data are within the manuscript and its Supporting Information files.*<br>• If neither of these applies but you are able to provide **details of access elsewhere**, with or without limitations, please do so. For example:<br><br>*Data cannot be shared publicly because of [XXX]. Data are available from the XXX Institutional Data Access / Ethics Committee (contact via XXX) for researchers who meet the criteria for access to confidential data.*<br><br>*The data underlying the results presented in the study are available from (include the name of the third party*	All relevant data are within the manuscript and its Supporting Information files.

and contact information or URL).<br>• This text is appropriate if the data are owned by a third party and authors do not have permission to share the data.<br><br>* typeset	
Additional data availability information:	

Powered by Editorial Manager® and ProduXion Manager® from Aries Systems Corporation

Manuscript Click here to access/download;Manuscript;Manuscript.docx

TYPE OF MANUSCRIPT: ORIGINAL RESEARCH ARTICLE

Mediating Role of Attitude on the Relationship Between Socio-Ecological Factors and Non-Communicable Disease Management among Support Staff in Putrajaya, Malaysia

Azrin Shah Abu Bakar[1], Haliza Abdul Rahman[1,2*], Ahmad Iqmer Nashriq Mohd Nazan[3]

[1] Department of Environmental and Occupational Health, Faculty of Medicine and Health Sciences, Universiti Putra Malaysia, 43400 Serdang, Selangor, Malaysia.
[2] Institute for Social Science Studies, Universiti Putra Malaysia, 43400 Serdang, Selangor, Malaysia.
[3]Department of Community Health, Faculty of Medicine and Health Sciences, Universiti Putra Malaysia, 43400 Serdang, Selangor, Malaysia.

***** dr.haliza@upm.edu.my

Mediating Role of Attitude on the Relationship Between Socio-Ecological Factors and Non-Communicable Disease Management among Support Staff in Putrajaya, Malaysia

ABSTRACT

Non-communicable disease (NCD) is a major global health issue that contributes to morbidity and mortality problems across countries including Malaysia. Low-income groups are one of the most vulnerable and at high-risk groups for NCD. However, no empirical study has dealt with the mediating effect of attitude between socio-ecological factors (intrapersonal, interpersonal, organizational, community and societal factors) and NCD management. Thus, this study aimed to examine the mediating role of attitude on the relationship between socio-ecological factors and NCD management. This study employs a cross-sectional survey of 453 support staff at selected ministries in Putrajaya, Malaysia. Data collected included socio-demographic information, knowledge of NCD, attitude towards preventing NCD, and Chronic Illness Resources Survey (CIRS). SPSS version 27 and Analysis of Moment Structures (AMOS) version 24 were used to analyze the data through the descriptive analysis and Structural Equation Model (SEM). The path analysis showed that attitude, community and societal factors effect on NCD management. Mediation effects showed a significant indirect effect of intrapersonal, community and societal factors on NCD management through attitude. These findings indicated that intrapersonal, community and societal factors are mediated by attitude, which impacts NCD management. This study suggests that the relevant stakeholders such as government, policymakers, health professionals and program managers pay attention to intrapersonal, community and societal factors to improve NCD management and enhance strategies, policies, and planning on NCD management among low-income groups in Malaysia.

Keywords: Attitude, Socio-ecological factors, Non-communicable Disease Management, Support staff, Mediation Effects, Malaysia

Introduction

Globalization, urbanization, and technological advancements have significantly contributed to the rise in non-communicable diseases (NCD) worldwide, Malaysia is also no exception in facing this issue. Despite the various preventive efforts made by the government through programs, activities and campaigns focusing on managing conditions of NCD. The trend of NCD in Malaysia continues increasing throughout the year. According to the National Health and Morbidity Survey (NHMS) in Malaysia, found that the prevalence of diabetes, hypertension, hypercholesterolemia, overweight and obesity remains high among the population [1]. Moreover, NHMS from 2015 to 2019 reported that there is an increasing trend of NCD such as diabetes and hypertension among low-income group compared to other income group [2.3]. This demonstrates that there is an increasing trend of NCD among the population exposing them to an increased risk of NCD.

The rise of NCD significantly impacts individuals' health, quality of life, and workforce productivity by causing increased absenteeism, decreased work performance, and the country's financial burden [4]. Indeed, the underprivileged are unable to access good health care and facilities, putting them at high risk for NCD [5]. Moreover, they also lose their source of family income to cover the cost of treatment and healthcare or the financial burden of NCD [6]. It seems the low-income group had the most impact on this issue.

Addressing NCD requires a comprehensive, efficient and cost-effective approach to improve health and well-being. Additionally, understanding the factors associated with NCD is important for developing health interventions for this vulnerable population. NCD management is one approach to enhance the prevention and control of NCD. It is also considered to be an effective low-cost initiative to prevent and manage NCD [7,8]. This is consistent with the study conducted by Lorber et al. (2024) reported that there was a

significant relationship between quality of life, general health and management of NCD. Therefore, NCD management is one of the best approaches to improving the quality of life and well-being [9].

Broadly, NCD is complex, whereby it requires engagement resulting from multiple contributors including individual, social, community and societal factors [10]. This is because personal, social, organizational, community and societal factors can positively or negatively impact an individual's influence behavior. Thereby, effective strategies for NCD management require the cooperation of multi-sectoral collaboration and action. The Socio-Ecological Model (SEM) is one of the most commonly used theories formulated by McLeroy et al. (1988) to explain environmental causes of health behavior, health promotion and identified interventions that offer five levels of influence specific to health behavior including intrapersonal, interpersonal, organizational, community, and societal factors [11]. In addition, SEM model can provide a good understanding of the various influences that affect health outcomes in reducing health inequality, especially in low-income groups [12].

Apart from that, attitude plays a crucial role in shaping human behavior and it can be considered to be a mediator of preventive health behaviors. Attitude is the evaluation, feelings, and tendencies towards objects, events, groups, or symbols, which significantly influence individuals to engage in actions that promote health and prevent diseases. Attitude can also drive motivation to initiate health-related behavioral actions. Individuals who have a positive attitude can influence engagement to behave towards the management of non-communicable diseases, while a negative attitude implies a low attitude evaluation can influence and hinder the development of good NCD management. This means that attitude is an important factor in shaping decisions that encourage a person to engage in preventive behavior. Therefore, an important emphasis on understanding predictors and mediating

attitude should be studied in determining behavior related to NCD. Nevertheless, there is still a limited number of studies on the health and preventive NCD of low-income group. Most studies on NCD among low-income groups have more focus on several factors such as demographic, socio-economic and health characteristics [6], prevalence of NCDs, socio-demographic and socio-economic factors [13,14], socio-demographic factors [15], demographic and socioeconomic factors [16], health status and living needs [17], health, mental health, health and nutritional behaviors [18] and quality of life [19]. Furthermore, there are still few local researchers who use the Socio-Ecological Model (SEM) to test the effect of attitude as a mediator in assessing socio-ecological factors (intrapersonal, interpersonal, organizational, community and societal). Moreover, the role of attitude as a mediator is still limited in studying a relationship [20]. Therefore, this study was conducted to examine the mediating role of attitude on the relationship between socio-ecological factors and NCD management among low-income workers in Malaysia.

Material and Methods

Study Design and Study Location

A cross-sectional study was conducted from 6 June to 30 August 2024 among support staff at selected ministries in Putrajaya. Putrajaya is located approximately 25 kilometers south of the capital Kuala Lumpur. It is the administrative center of the Malaysian government, where it has replaced Kuala Lumpur as the Government Administrative Center to avoid congestion around the Kuala Lumpur area. Putrajaya is a planned city consisting of ministries, housing for civil servants, lakes and green areas to provide peace from the stress of modern life. Thus, this location was chosen because it is the Federal Government Administration Center which represents the largest number of civil servants in Malaysia.

Sampling Technique and Sample

A cluster random sampling technique was used to recruit participants. From 24 ministries, 8 ministries were randomly selec[illegible] Then, all support staff who met the inclusion criteria were invited to participate in this study. The permanent support staff aged between 18-60 years old were recruited into the study, exclusion criteria were contract or part-time staff and those who refused to participate.

Sam[illegible]ize

Sample size based on an online calculator for Structural Equation Modeling (SEM) of Soper, D.S. (2020) by setting the anticipated effect size f2 = 0.3, the desired p-value was at 0.05, the desired statistical power level at 80%, the number of observed variables was 94 and the number of latent variables was 12, the number of minimum sample sizes suggested is 200 [21]. According to Hair et al. (2010) suggest that sample size should be appropriate for SEM in the range of 100 to 400 subjects. Hence, this study took 400 samples and adjusted for a 20% non-response rate, the minimum sample size required in this study was 480 [22].

Instrume[illegible]

This study instrument comprises four sections which include socio-demographic information, knowledge of NCD, attitude towards preventing NCD, and Chronic Illness Resources Survey (CIRS). The questionnaire was modified and adapted from previous st[illegible]es [23,24]. Section A consists of socio-demographic information including age, gender, race, marital status, educational level, and monthly household income. Section B a[illegible] the respondents' knowledge of NCD, The knowledge questions cover the understanding of NCD including the major NCD such as cardiovascular disease, stroke and hypertension, diabetes mellitus, and chronic obstructive pulmonary disease as well as risk factors, complications and disease management. Respondents were instructed to give a ‘yes’, ‘n[illegible]nd ‘do not know’. [illegible] knowledge level was categorized as poor for scores within 0-50%, for scores within 51-

75% was considered moderate, and good for scores within 76-100%. It had excellent internal consistency reliability (Cronbach's α= 0.961). Section C consists of 15 items for the attitude towards preventing NCD which include balanced diet, physical activity, smoking, salt intake, medication intake, and treatment checks. The question evaluated using a 5-point Likert scale ranging from 'Strongly disagree', 'disagree', 'agree' and 'strongly agree'. The total score was divided into three categories as follows: poor attitude for scores (0-50%), moderate attitude for scores (51-75%) and good attitude for scores (76-100%). In this study, the survey showed good internal consistency reliability (Cronbach's α= 0.898). Section D was the final section of the questionnaire containing 65 items for Chronic Illness Resources Survey (CIRS) to assess the socio-ecological factors, divided into five sub-scales that measure interpersonal, organizational, community, societal and management of disease. The questionnaire is based on a 5-point Likert scale as either 'Not at all', 'A little', 'Moderate', 'Quite a lot' and 'A great deal'. The total score can be categorized as follows: less than 60% was considered low, 60-80% was considered moderate, and 80% and above was considered high. In this study, the CRIS showed good internal consistency reliability (Cronbach's α= 0.90).

Ethics considerations

Ethical approval to conduct this study was obtained from the Ethics Committee for Research Involving Human Subjects, Universiti Putra Malaysia with the reference number of JKEUPM-2024-301. In order to assist the respondents in understanding the reason for this study, the respondents were informed about the study, and written informed consent was obtained from respondents before participating the study. Confidentiality of the information were maintained in strictest confidence as well as name and identifying information were kept anonymous upon data analysis.

Data Analysis

Data analysis was performed using SPSS version 27 and Analysis of Moment Structure (AMOS) 24. Descriptive analysis was performed by using frequencies and percentages. Structural Equation Model (SEM) analysis was used to examine the interrelationship between the constructs in the hypothesized model. Then, a test of a mediation effect was done through a bootstrap analysis. The significance level was set at $p < 0.05$.

Results

Socio-demographic characteristics

A total of 530 support staff were invited to participate in this study. Only 458 participated, giving the response rate of 85%. The socio-demographic characteristics are shown in Table 1. The majority were between 30-39 y[illegible] old, female (73.1%), Malay (94.9%) and married (75.7%),. The household income of all the respondents' range between RM2501-RM3000. Less than half of [illegible]n (42.4%) had STPM/ Matriculation/ Diploma. Around (95.8%) reported did not have NCD.

Table 1. Socio-demographic characteristics of the respondents

Socio-demographic Characteristics	Frequency (n)	Percentage (%)
**Age**		
Below than 30 years old	22	4.9
30 - 39 years old	242	53.4
40 - 49 years old	172	38.0
50 - 59 years old	17	3.8
**Gender**		
Male	122	26.9
Female	331	73.1
**Ethnicity**		
Malay	430	94.9
Chinese	1	0.2
Indian	6	1.3
Others	16	3.5

**Marital Status**		
Single	89	19.6
Married	343	75.7
Widowed/Divorced/Separated	21	4.6
**Monthly Household Income**		
RM1001 - RM1500	26	5.7
RM1501 - RM2000	67	14.8
RM2001 - RM2500	88	19.4
RM2501 - RM3000	107	23.6
RM3001 - RM3500	72	15.9
RM3501 - RM4000	48	10.6
More than RM4000	45	9.9
**Educational Level**		
Degree	104	23.0
STPM/ Matriculation/ Diploma	192	42.4
SPM/ Certificate	154	34.0
PT3/ PMR/ SRP	3	0.7
**Presence of chronic disease**		
No	434	95.8
Yes	19	4.19

Structural Equation Modeling Analysis

Based on SEM, the resulting structural model fits indicated the data fit the model with; [$\chi 2/df = 2.491$, P = 0.000, CFI = 0.921, TLI = 0.913, RMSEA = 0.057]. The value of the relative chi-square ($\chi 2/df$) was well below 5, indicating an acceptable fit between the proposed measurement model and the collected data. Other fit indices, namely CFI, and TLI values, were above the minimum value of .90, and the RMSEA value was below the cut-off value of .08 (RMSEA = .050). Therefore, the analysis of the SEM showed that the overall value of the fit index reached acceptable values and confirmed that the structural model was fit.

Causal path analysis

Table 2 shows the results of the influence intrapersonal factor (knowledge), interpersonal factors (family and friends), organizational factor (workplace psychosocial support),

community factors (neighborhood, local community, community organization and healthcare services) and societal factors (health policy of healthcare financing and mass media) on NCD management were assessed through path analysis. Attitude (β = 0.450, p = 0.000), community factors (β = 0.443, p = 0.000), and societal factors (β = 0.366, C.R.= 5.360, p = 0.000) showed significant effect on NCD management. This indicated that community factors, societal factors and attitude significantly influence~~s~~ NCD management.

Table 2. Regression Weights in Direct Hypothesize Model

Construct	Path	Construct	Estimate	S.E.	C.R.	P
Non-Communicable Disease Management	<---	Attitude	0.45	0.123	3.655	0.000
Non-Communicable Disease Management	<---	Intrapersonal Factor	0.001	0.002	0.325	0.745
Non-Communicable Disease Management	<---	Interpersonal Factors	-0.038	0.068	-0.562	0.574
Non-Communicable Disease Management	<---	Organizational Factors	-0.002	0.052	-0.04	0.968
Non-Communicable Disease Management	<---	Community Factors	0.443	0.055	8.087	0.000
Non-Communicable Disease Management	<---	Societal Factors	0.366	0.068	5.36	0.000

Note: S.E: Standard Error; C. R: Critical Ratio for regression weight; P: *** < 0.001

Test of mediation effects

The bootstrapping procedure was conducted to test mediation effects. Table 3 shows the results of attitude mediates the relationship between intrapersonal, interpersonal, organizational, community and societal factors with NCD management. These findings revealed that the indirect effect of intrapersonal, community and societal factors on NCD management through the mediator of attitude was statistically significant at the .05 level of significant. In explaining the mediation effect, the findings show significant effects were detected for community factors in direct model (Beta = 0.514, p = 0.001) and the indirect model (Beta = 0.054 p = 0.005). Likewise, there were statistically significant effects of societal factors, both in the direct model (Beta = 0.329, p = 0.005), as well as in the indirect model (Beta = 0.041, p < 0.010). Therefore, it could be concluded that attitude partially mediated the relationship between community and societal factors on NCD management. Nevertheless, direct effect of intrapersonal factor (knowledge) with NCD management was not statistically significant (β = 0.01, p = 0.625). When attitude was included as a mediator, the results show that intrapersonal factor (knowledge) on NCD management was significant (β = 0.016, p =0.015). These findings indicated that attitude fully mediated the relationship between intrapersonal factor (knowledge) on NCD management.

Table 3. Results of bootstrap analysis

Model/Hypothesized Path	Beta	P	95% CI BC		Result
			Lower	Upper	

			Bound	Bound	
**Direct Model:**					
Intrapersonal Factor → Non-Communicable Disease Management	0.01	0.625	-0.045	0.077	Not Significant
**Indirect Model:**					
Intrapersonal Factor → Attitude → Non-Communicable Disease Management	0.016	0.015	0.002	0.046	Significant
**Direct Model:**					
Interpersonal Factors → Non-Communicable Disease Management	-0.029	0.612	-0.162	0.085	Not Significant
**Indirect Model:**					
Interpersonal Factors → Attitude → Non-Communicable Disease Management	-0.010	0.186	-0.039	0.008	Not Significant

**Direct Model:**					
Organizational Factor → Non-Communicable Disease Management	-0.002	0.985	-0.090	0.099	Not Significant
**Indirect Model:**					
Organizational Factor → Attitude → Non-Communicable Disease Management	0.003	0.716	-0.013	0.033	Not Significant

**Direct Model:**					
Community Factors → Non-Communicable Disease Management	0.514	0.001	-0.351	0.652	Significant
**Indirect Model:**					
Community Factors → Attitude → Non-Communicable Disease Management	0.054	0.005	0.115	0.033	Significant
**Direct Model:**					
Soceital Factors → Non-Communicable Disease Management	0.329	0.005	-0.045	0.077	Significant
**Indirect Model:**					
Societal Factors → Attitude → Non-Communicable Disease Management	0.041	0.010	0.008	0.088	Significant

Discu[illegible]n

This study examines the mediating role of attitude on relationship between socio-ecological factors (intrapersonal, interpersonal, organizational, community and societal factors) with NCD management among support staff in Putrajaya, Malaysia. This result shows that attitude, community and social factors influence NCD management. Attitude exerted the greatest direct influence on non-communicable disease management. This may be due to having a good attitude, individuals are confident in practice towards preventing chronic disease [25]. This indicates that a more positive attitude leads to stronger NCD management. Therefore, when individuals have a positive attitude, it helps to improve their management of non-communicable disease. Additionally, findings revealed that community factors (neighborhood, local community, community organization and healthcare services) influence NCD management. It indicated that community factors effective influence NCD management among respondents. One probable explanation is that the approach of health promotion programs applied in the community has increased their awareness of NCD. This result was consistent with the previous studies, which have reported that community-based programs such as Komuniti Sihat Perkasa Negara (KOSPEN) help deal with NCD and prevent and control NCD risk factors [26]. Another study found that community engagement involves partnerships with local health organizations such as community outreach programs and hosting health awareness events contribute to the overall well-being of the communities [27]. Therefore, the involvement of community health workers as the health care team is one of the good efforts of the community program in increasing access to treatment and health care. This indicated that the initiative of the community approach helps ongoing healthcare

with prevention and control of NCD risk occurring in the community [28]. Another possible reason is that Putrajaya was designed with a neighborhood concept in the precinct as a garden city concept town, which provides neighborhood parks such as green spaces, gardens and lakes to the residents, which promotes physical activity (e.g., walking, jogging and cycling) . A previous study found that open spaces in Putrajaya significantly contribute to health promotion [29]. A similar finding was also reported that the presence of green urban has promoted healthy communities and healthy lifestyles [30]. Thus, it encourages a healthier lifestyle through community engagement in outdoor activities with community bonds through shared recreational spaces. Besides that, since the respondents live in an urban area, the majority of them have easy access to healthcare services. Urban areas make it easier for people to access healthcare services due to better public transportation options and a variety of clinic and hospital services, which influence management of non-communicable diseases. In fact, individuals from relatively low and middle income prefer to choose services from their community, where they need only pay MYR 1 for a general outpatient consultation and MYR 5 for a specialist consultation [31]. This is consistent with the report by NHMS 2019 revealed that 8.1% of B40 households in Malaysia have received healthcare services as outpatients in the last two weeks prior from healthcare professionals [2]. Moreover, the government's role at the primary care level in NCD management has seen an increase in the role of doctors, nurses and medical assistants, where follow-up care is done at the many primary health clinics [32]. This is supported by [33] mention that management in the primary care system provides services to the community for good disease management, patient empowerment and subsequently better health status among NCD patients as well as the general community. Furthermore, healthcare services help to prevent and cure health problems for the

public, which influences the outcomes of the population’s health status. Therefore, community factors with access to healthcare facilities, availability of recreational spaces and community health programs were effects on NCD management among support staff.

This study also revealed that societal factors (health policy of healthcare financing and mass media) were identified as significant factors that significantly influenced NCDM. This could be a health policy in health care financing by the government helping protect them from NCD.This is in line with the findings of the NHMS (2009-2023) which reported that government spending on health, especially on the low-income groups, reflects a strong public role in health financing to the low-income group. For example, PeKa B40 aims to improve access to healthcare services for low-income groups [34]. In addition, heavily subsidized by the government, providing affordable and accessible care to all citizens, especially low-income groups. Therefore, government healthcare financing plays a significant role in protecting populations, especially low-income groups. On the other that, mass media also play an important role in influencing NCD management. The existence of mass media provides useful benefits to the public and makes it easier for them to get information, share information and exchange existing information which further influences healthy behavior. The findings are consistent with prior research indicating that social media influences preventive behavior through awareness and information exchange [35]. Another study found that mass media use influences health behavior [36]. This implies that NCD management can be improved by enhancing attitude, community and societal factors.

The current study showed that attitude mediates the relationship between intrapersonal, community and societal factors on NCD management. This study found that community and societal factors were partially mediated by attitude. The partial mediating role of attitude was due

to community and societal factors having direct and indirect relationships with NCD management through attitudes as mediators. Additionally, attitude fully mediates the relationship between intrapersonal factor of knowledge and NCD management. This indicated that attitude helps to regulate the relationship between intrapersonal, community and societal factors toward NCD management. In other words, improvement in intrapersonal, community factors, and societal factors encouraged individuals to attitude better, which further enhanced NCD management as well. Furthermore, by increasing an individual's level of attitude, their build practices toward the prevention of NCD can be achieved. This finding of this study were consistent with previous studies, which proved attitude as a mediator in controlling the relationship between exogenous variables and endogenous variable [37-39]. Hence, attitude as a mediator has an impact to interpersonal, community factors societal factors on NCD management. Taken together, relevant stakeholders might better consider mediated relationships in future intervention programs to promote awareness and understanding of NCD towards achieving NCD management.

Conclusion

Attitude, community and societal factors have direct effects on NCD management based on the results of the SEM analysis. The bootstrapping test found that attitude act as a partial mediator in the relationship between community and societal factors towards NCD management, while attitude fully mediates the relationship between intrapersonal factor towards NCD management. These findings provide valuable insights to the relevant stakeholders such as government, policymakers, health professionals and program managers to address NCD management among support staff and highlight the importance of further research on NCDM by paying attention

through mediated relationships and proposed framework through education and promotion of health and prevention practices against NCD by providing the development of intervention programs in the future. However, the study has limitations such as the use of the self-report questionnaires, it’s possible gave a false or incomplete response. Additionally, the cross-sectional design impossible to establish causality, whereby longitudinal studies could help determine. Furthermore, this study was focused on Putrajaya, Malaysia. Therefore, it is suggested that future studies could include in different locations. The scope of the study can be also expanded to include other low-income workers (e.g., private sector employees and nongovernmental organizations (NGOs) to reflect the whole population of low-income workers in Malaysia.

References

1. Institute of Public Health, Technical Report National Health and Morbidity Survey (NMHS) 2023. Non-Communicable Disease and Healthcare Demand, Malaysia, 2024.

2. Institute of Public Health, National Health and Morbidity Survey 2019 (NHMS 2019). Non-communicable diseases, healthcare demand, and health literacy. Kuala Lumpur: Ministry of Health Malaysia, 2019.

3. Institute of Public Health, National Health and Morbidity Survey 2015 (NHMS 2015). Vol. II: Non-Communicable Diseases, Risk Factors & Other Health Problems. Kuala Lumpur: Ministry of Health Malaysia, 2015.

4. Budreviciute A, Damiati S, Sabir DK, Onder K, Schuller-Goetzburg P, Plakys G, , et al. Management and prevention strategies for non-communicable diseases (NCDs) and their risk factors. Front. public health. 2020; 8:574111. https://doi: 10.3389/fpubh.2020.574111.

5. Stringhini S, Carmeli C, Jokela M, Avendaño M, Muennig P, Guida F,et al., Socioeconomic status and the 25× 25 risk factors as determinants of premature mortality: a multicohort study and meta-analysis of 1· 7 million men and women. Lancet. 2017; 389 (10075):1229–1237. https://doi.org/10.1016/S0140-6736(16)32380-7.

6. Isamail N, Said R, Ismail NW, Haron SA. Non-Communicable Diseases Impact Low-Income Households in Malaysia. Malaysian Journal of Medical Sciences. 2024;31(1): 124. https://doi: 10.21315/mjms2024.31.1.11.

7. PhillipsCM, Chen LW, Heude B, Bernard JY, Harvey NC, Duijts L, et al. Dietary inflammatory index and non-communicable disease risk: a narrative review. Nutrients. 2019;11(8): 1873. https://doi: 10.3390/nu11081873.

8. Lee IM, Shiroma EJ, Lobelo F, Puska P, Blair SN, Katzmarzyk PT. Effect of physical inactivity on major non-communicable diseases worldwide: an analysis of burden of disease and life expectancy. Lancet. 2012;380 (9838): 219 –229. https://doi: 10.1016/S0140-6736(12)61031-9.

9. Lorber M, Reljić NM, Kegl B, Fekonja Z, Štiglic G, Davey A, et al. Person-Centred Care: A Support Strategy for Managing Non-Communicable Diseases. Healthcare. (2024);12(5): 526. https://doi.org/10.3390/healthcare12050526.

10. Nguyen KH, Fields JD, Cemballi AG, Desai R, Gopalan A, Cruz T, et al. The role of community-based organizations in improving chronic care for safety-net populations. Journal of the American Board of Family Medicine. 2021;34 (4):698-708, https://doi: 10.3122/jabfm.2021.04.200591.

11. McLeroy KR, Bibeau D, Steckler A, Glanz K. An Ecological Perspective on Health Promotion Programs. Health Educ. Q. 1988; 315(4):51–377. https://doi: 10.1177/109019818801500401.

12. Baron SL, Beard S, Davis LK, Delp L, Forst L, Kidd-Taylor A,, et al. Promoting Integrated Approaches to Reducing Health Inequities Among Low- Income Workers: Applying a Social Ecological Framework. Am. J. Ind. Med. 2014;57: 539–556. https://doi: 10.1002/ajim.22174.

13. Hami R, Shahabudin SA, Shariff NM, Mohd Mujar NM, Isa S, Roslan H, et al. Factors Contributing to Non-Communicable Diseases in a Selected Low Socio-Economic Status Community in Kedah, Malaysia. Malaysian Journal of Medicine & Health Sciences. 2021;17(1): 117–123.

14. Harris H, Ooi YB, Lee JS, Matanjun P. Non-communicable diseases among low income adults in rural coastal communities in Eastern Sabah, Malaysia. BMC Public Health. 2019;19: 1–13. https://doi.org/10.1186/s12889-019-6854-6.

15. Fiidow OA, Huda BZ, Salmiah MS. Salmiah, Socio-demographic factors of hypertension among non-academic staff in Universiti Putra Malaysia. International Journal of Public Health and Clinical Sciences. 2016;3(4):158 –168.

16. SuTT, Amiri M, Mohd Hairi F, Thangiah N, Bulgiba A, Majid HA. Prediction of Cardiovascular Disease Risk among Low-Income Urban Dwellers in Metropolitan Kuala Lumpur, Malaysia. BioMed Res Int. 2015;2015(1): 516984. https:// doi: 10.1155/2015/516984.

17. Puteh SE, Kadir HA.. Factors associated with health status and living needs impact on b40 women's quality of life in Malaysia: an analysis based on b40 rural and urban poor areas. Malaysian J. Public Health Med. 2022; 22(2):176–186. https://doi.org/10.37268/mjphm/vol.22/no.2/art.1717.

18. Shahar S, Lau H, Puteh SE, Amara S, Razak NA. Health, access and nutritional issues among low-income population in Malaysia: Introductory note. BMC Public Health. 2019;19: 1–5. https://doi.org/10.1186/s12889-019-6852-8.

19. Wan PutehSE, Siwar C, Zaidi MA, Abdul Kadir H. Health related quality of life (HRQOL) among low socioeconomic population in Malaysia. BMC Public Health. 2019;19: 1–8. https://doi.org/10.1186/s12889- 019-6853-7.

20. ÇabukS, Tanrikulu C, Gelibolu L. Understanding organic food consumption: attitude as a mediator. International Journal of Consumer Studies. 2014;38: 337–345. https://doi.org/10.1111/ijcs.12094.

21. Soper DS, A-Priori Sample Size Calculator for Structural Equation Models [Software]. http://wwwdanielsopercom/statcalc, 2020.

22. Hair F, Black WC, Anderson RE. Multivariate Data Analysis. (7th Ed). New York: Pearson. 2010.

23. Ithnin M, Nor NA, Nordin NJ, Effendy NM, Sahar MA, Abdullah KH,, et al. Knowledge, attitude, and practice on Non- Communicable Diseases (NCDs) among the adult population in the urban area of Negeri Sembilan, Malaysia. International Journal of Research in Pharmaceutical Sciences. 2018; 9 (2Suppl): 88–94. https://doi.org/10.1080/14635240.2020.1749526.

24. Glasgow RE, Strycker LA, Toobert DJ, Eakin E. A Social-Ecologic Approach to Assessing Support for Disease Self- Management: The Chronic Illness Resources Surveys. J. Behav. Med. 2000;23(6): 559-583. https:// doi:.10.1023/a:1005507603901.

25. Ithnin M, Nor NA, Juliana N, Effendy NM, Sahar MA, Abdullah KH, et al. Knowledge, Attitude and Practices Towards Lifestyle Related Non-Communicable Diseases (NCDs): A Cross

Sectional Study among Indigenous Orang Asli Adults in Negeri Sembilan, Malaysia. IIUM Medical Journal Malaysia. 2020;19 (2):75–82. https://doi.org/10.31436/imjm.v19i2.1569.

26. Lim KH, M Fadhli Y, Omar M, Rosnah R, M. Nazaruddin B, Sumarni MG, et al. Evulation of Effectiveness of Implementation of “Komuniti Sihat Perkasa Negara (KOSPEN) Programme in Malaysia- Phase 1. Kuala Lumpur: Ministry of Health Malaysia, 2015.

27. Kabuya KE. Influence of Workplace Health on Awareness of Non- Communicable Diseases (NCDs) and Its Cascading Effects on the Well-being of Families and Communities. International Journal of Sciences: Basic and Applied Research. 2024. 73 (1):43–58.

28. Kataria I, Ngongo C, Lim SC, Kocher E, Kowal P, Chandran A, et al. Development and evaluation of a digital, community-based intervention to reduce noncommunicable disease risk in a low-resource urban setting in Malaysia: a research protocol. Implementation science communications. 2020;1: 1–9. https://doi.org/10.1186/s43058-020-00080-y.

29. Danjaji AS, Danladi M, Adamu A, Danladi HM. Urban Green Spaces as Promoters of Healthy Living: Evidence from Putrajaya, Malaysia. Research Square. 2021: 1 –17. https://doi.org/10.21203/rs.3.rs-1166766/v1.

30. Ayob Z, A.R. Amir AR. Developing Urban Walkability Measures Grounded in Green Urbanism Principles Using The Delphi Survey Study. Planning Malaysia. 2024; 22:307–320.

31. Aizuddin AN, Al Junid SM. Willingness to pay for outpatient services user fees: Malaysian community perspective. Jurnal Sains Kesihatan Malaysia. 2018; 16(1):145-153.

32. Mustapha FI, Omar ZA, Mihat O, Md Noh K, Hassan N, Abu Bakar R, et al. Addressing non-communicable diseases in Malaysia: an integrative process of systems and community. BMC Public Health. 2014;14: 1–6. htttps://doi:doi: 10.1186/1471-2458-14-S2-S4.

33. Fadzilah A, Azima R, Khalsom S, Rosliza AM. Governance framework in non-communicable disease (NCD) control and prevention programme at primary care level in Malaysia. International Journal of Public Health and Clinical Sciences. (2020); 7(3):167-185.

34. Yunus SZ, Puteh SE, Ali AM, Daud F., S.E.W. Puteh, A.M. Ali, F. Daud, The Covid impact to public healthcare utilization among urban low-income subsidized community in Klang Valley Malaysia. Health Serv. Res. Manag, Epidemiol. 2021;8: 1–9. https://doi: 10.1177/23333928211002407.

35. Nazir M, Hussain I, Tian J, Akram S, Mangenda Tshiaba S, Mushtaq S, et al. A multidimensional model of public health approaches against COVID-19. International Journal of Environmental Research and Public Health. 2020; 17(11): (3780. https://doi.org/10.3390/ijerph17113780.

36. Nabi RL, Huskey R, Nicholls SB, Keblusek L, Reed M. When audiences become advocates: Self-induced behavior change through health message posting in social media. Computers in Human Behavior. 2019;99: 260–267. https://doi.org/10.1016/j.chb.2019.05.030.

37. Zhang Y, Zhang B, Chen C, Feng X, Song S, Wang H. The mediation effect of attitude on the association between knowledge and self-management behaviors in Chinese patients with diabetes. International Journal of Public Health. 2023; 68: 1606022. https;//doi: 10.3389/ijph.2023.1606022.

38. Saengcha S, Pattanapongthorn J, Jermsittiparsert K. Influence of Beliefs on Attitudes and Intention of Adopting e-Health System among the Public Hospitals of Thailand. International Journal of Innovation, Creativity and Change. 2019; 8(8):238–255.

39. An HG, Ha JY, Kim SH. Mediating Effect of Attitude on the Relationship between Knowledge about Tuberculosis and Preventive Behavior Toward Tuberculosis of Foreign Students.

International Journal of Bio-Science and Bio-Technology. 2016;8(2):181–196. https://dx.doi.org/10.14257/ijbsbt.2016.8.2.17.

Click here to access/download
Supporting Information
Questionnaire.docx
