# Supplementary material for: Determinants of non-communicable disease management among support staff in Putrajaya, Malaysia: The mediating role of attitude
Source: PLoS One. 2026 Apr 30;21(4):e0347528. doi: 10.1371/journal.pone.0347528 (PMC13132246; doi:10.1371/journal.pone.0347528)
Supplement: Attachment — Submitted filename: PONE-D-25-10747_R1_reviewer ZAK020126.pdf [file pone.0347528.s005.pdf]

PLOS ONE

Mediating Role of Attitude on the Relationship Between Socio-Ecological Factors and Non-Communicable Disease Management among Support Staff in Putrajaya, Malaysia

--Manuscript Draft--

**Manuscript Number:**	PONE-D-25-10747R1
**Article Type:**	Research Article
**Full Title:**	Mediating Role of Attitude on the Relationship Between Socio-Ecological Factors and Non-Communicable Disease Management among Support Staff in Putrajaya, Malaysia
**Short Title:**	Mediating Role of Attitude on the Relationship Between Socio-Ecological Factors and NCD Management
**Corresponding Author:**	Haliza Abdul Rahman, PhD<br>Universiti Putra Malaysia<br>Serdang, Selangor MALAYSIA
**Keywords:**	Attitude; socio-ecological factors; Non-communicable Disease Management; Support staff; Malaysia
**Abstract:**	Non-communicable disease (NCD) is a major global health issue that contributes to morbidity and mortality problems across countries including Malaysia. The social and environment are increasingly recognized as a critical factor shaping the development of NCDs, particularly among vulnerable groups such as low-income households in managing chronic diseases. However, there was still limited information regarding the relationship between socio-ecological factors (i.e., intrapersonal, interpersonal, organizational, community and societal factors) on NCD management through attitude. Thus, this study aimed to examine the mediating role of attitude on the relationship between socio-ecological factors and NCD management. This study employs a cross-sectional survey of 200 support staff with NCD at selected ministries in Putrajaya, Malaysia. Data collected included socio-demographic information, knowledge of NCD, attitude towards NCD, and Chronic Illness Resources Survey (CIRS). SPSS version 27 and Analysis of Moment Structures (AMOS) version 24 were used to analyze the data through the descriptive analysis and Structural Equation Model (SEM). The path analysis showed that attitude, community and societal factors affect NCD management. Furthermore, medication analysis revealed that attitude mediates the relationship between intrapersonal, community and societal factors and NCD management. This study suggests that the relevant stakeholders such as policymakers, healthcare providers, and program managers should pay attention to intrapersonal, community and societal factors through the implementation of strategies, policies and planning as well as designing holistic interventions to improve NCD management.
**Order of Authors:**	Haliza Abdul Rahman, PhD
	Azrin Shah Abu Bakar
	Ahmad Iqmer Nashriq Mohd Nazan, PhD
**Response to Reviewers:**	I would like to express my gratitude to the reviewers for their helpful criticisms and comments, which have enabled us to improve the quality of this manuscript.<br><br>Please refer to the manuscript highlighted in blue for our responses to each of the reviewers' comments.<br><br>Thank you.
**Additional Information:**	
**Question**	**Response**
**Financial Disclosure**<br><br>Enter a financial disclosure statement that describes the sources of funding for the	The author(s) received no specific funding for this work.

work included in this submission. Review the submission guidelines for detailed requirements. View published research articles from *PLOS ONE* for specific examples.<br><br>This statement is required for submission and **will appear in the published article** if the submission is accepted. Please make sure it is accurate.<br><br>**Funded studies**<br>Enter a statement with the following details:<br>• Initials of the authors who received each award<br>• Grant numbers awarded to each author<br>• The full name of each funder<br>• URL of each funder website<br>• Did the sponsors or funders play any role in the study design, data collection and analysis, decision to publish, or preparation of the manuscript?<br><br>Did you receive funding for this work?	
**Competing Interests**<br><br>Use the instructions below to enter a competing interest statement for this submission. On behalf of all authors, disclose any competing interests that could be perceived to bias this work—acknowledging all financial support and any other relevant financial or non-financial competing interests.<br><br>This statement is required for submission and **will appear in the published article** if the submission is accepted. Please make sure it is accurate and that any funding sources listed in your Funding Information later in the submission form are also declared in your Financial Disclosure statement.<br><br>View published research articles from *PLOS ONE* for specific examples.	The authors have declared that no competing interests exist.

**NO authors have competing interests**<br><br>Enter: *The authors have declared that no competing interests exist.*<br><br>**Authors with competing interests**<br><br>Enter competing interest details beginning with this statement:<br><br>*I have read the journal's policy and the authors of this manuscript have the following competing interests: [insert competing interests here]*<br><br>* typeset	
**Ethics Statement**<br><br>Enter an ethics statement for this submission. This statement is required if the study involved:<br><br>• Human participants<br>• Human specimens or tissue<br>• Vertebrate animals or cephalopods<br>• Vertebrate embryos or tissues<br>• Field research<br><br>Write "N/A" if the submission does not require an ethics statement.<br><br>General guidance is provided below. Consult the submission guidelines for detailed instructions. **Make sure that all information entered here is included in the Methods section of the manuscript.**	Ethical approval to conduct this study was obtained from Ethics Committee for Research Involving Human Subject, Universiti Putra Malaysia with the reference number of JKEUPM-2024-301.

**Format for specific study types**<br><br>**Human Subject Research (involving human participants and/or tissue)**<br>• Give the name of the institutional review board or ethics committee that approved the study<br>• Include the approval number and/or a statement indicating approval of this research<br>• Indicate the form of consent obtained (written/oral) or the reason that consent was not obtained (e.g. the data were analyzed anonymously)<br><br>**Animal Research (involving vertebrate animals, embryos or tissues)**<br>• Provide the name of the Institutional Animal Care and Use Committee (IACUC) or other relevant ethics board that reviewed the study protocol, and indicate whether they approved this research or granted a formal waiver of ethical approval<br>• Include an approval number if one was obtained<br>• If the study involved *non-human primates*, add *additional details* about animal welfare and steps taken to ameliorate suffering<br>• If anesthesia, euthanasia, or any kind of animal sacrifice is part of the study, include briefly which substances and/or methods were applied<br><br>**Field Research**<br><br>Include the following details if this study involves the collection of plant, animal, or other materials from a natural setting:<br>• Field permit number<br>• Name of the institution or relevant body that granted permission	
**Data Availability**<br><br>Authors are required to make all data underlying the findings described fully available, without restriction, and from the time of publication. PLOS allows rare exceptions to address legal and ethical concerns. See the PLOS Data Policy and FAQ for detailed information.	No - some restrictions will apply

A Data Availability Statement describing where the data can be found is required at submission. Your answers to this question constitute the Data Availability Statement and **will be published in the article**, if accepted.<br><br>**Important:** Stating 'data available on request from the author' is not sufficient. If your data are only available upon request, select 'No' for the first question and explain your exceptional situation in the text box.<br><br>Do the authors confirm that all data underlying the findings described in their manuscript are fully available without restriction?	
**Describe where the data may be found in full sentences. If you are copying our sample text, replace any instances of XXX with the appropriate details.**<br><br>• If the data are **held or will be held in a public repository**, include URLs, accession numbers or DOIs. If this information will only be available after acceptance, indicate this by ticking the box below. For example: *All XXX files are available from the XXX database (accession number(s) XXX, XXX.*).<br>• If the data are all contained **within the manuscript and/or Supporting Information files**, enter the following: *All relevant data are within the manuscript and its Supporting Information files.*<br>• If neither of these applies but you are able to provide **details of access elsewhere**, with or without limitations, please do so. For example:<br><br>*Data cannot be shared publicly because of [XXX]. Data are available from the XXX Institutional Data Access / Ethics Committee (contact via XXX) for researchers who meet the criteria for access to confidential data.*<br><br>*The data underlying the results presented in the study are available from (include the name of the third party*	The datasets used and analysed during the current study are available from the corresponding author upon reasonable request. Access to the data is subject to ethical approval and the confidentiality of study participants.

Powered by Editorial Manager® and ProduXion Manager® from Aries Systems Corporation

and contact information or URL).<br>• This text is appropriate if the data are owned by a third party and authors do not have permission to share the data.<br><br>* typeset	
Additional data availability information:	

Powered by Editorial Manager® and ProduXion Manager® from Aries Systems Corporation

Manuscript Click here to access/download;Manuscript;Manuscript..docx

TYPE OF MANUSCRIPT: ORIGINAL RESEARCH ARTICLE

Mediating Role of Attitude on the Relationship Between Socio-Ecological Factors and Non-Communicable Disease Management among Support Staff in Putrajaya, Malaysia

Azrin Shah Abu Bakar[1], Haliza Abdul Rahman[1,2*], Ahmad Iqmer Nashriq Mohd Nazan[3]

[1] *Department of Environmental and Occupational Health, Faculty of Medicine and Health Sciences, Universiti Putra Malaysia, 43400 Serdang, Selangor, Malaysia.*
[2] *Institute for Social Science Studies, Universiti Putra Malaysia, 43400 Serdang, Selangor, Malaysia.*
[3] *Department of Community Health, Faculty of Medicine and Health Sciences, Universiti Putra Malaysia, 43400 Serdang, Selangor, Malaysia.*

***** dr.haliza@upm.edu.my

Mediating Role of Attitude on the Relationship Between Socio-Ecological Factors and Non-Communicable Disease Management among Support Staff in Putrajaya, Malaysia

ABSTRACT

Non-Communicable Disease (NCD) is a major global health issue that contributes to morbidity and mortality problems across countries including Malaysia. Social environment is increasingly recognized as critical factors in shaping the development of NCDs that affect particularly vulnerable groups, especially low-income households, in managing chronic diseases. However, there was still limited information regarding the relationship between socio-ecological factors (i.e., intrapersonal, interpersonal, organizational, community, and societal factors) and NCD management through attitude. Therefore, this study aimed to examine the mediating role of attitude on the relationship between socio-ecological factors and NCD management. This study employs a cross-sectional survey of 200 support staff with NCD at selected ministries in Putrajaya, Malaysia. The data collected included socio-demographic information, knowledge of NCDs, attitudes towards NCDs, and the Chronic Illness Resources Survey (CIRS). Statistical Package of Social Sciences (SPSS) version 27 and Analysis of Moment Structures (AMOS) version 24 were employed to analyze the data through descriptive analysis and Structural Equation Model (SEM). The path analysis revealed that attitude, community, and societal factors affect NCD management. Further, mediation analysis indicated that attitude mediates the relationship between intrapersonal, community, and societal factors and NCD management. This study suggests that relevant stakeholders, including policymakers, healthcare providers, and program managers should pay attention to intrapersonal, community, and societal factors. This can be achieved through the implementation of strategies, policies, and planning, as well as designing holistic interventions to improve NCD management.

Keywords: Attitude, Socio-ecological factors, Non-communicable Disease Management, Support staff, Putrajaya, Malaysia

Introduction

Globalization, urbanization, technological advancements, and demographic (population aging) have contributed significantly to the increase in Non-Communicable Diseases (NCDs) in Malaysia. Despite various efforts undertaken by the government including programs, activities, campaigns, health screenings, and community participation in health promotion focus on managing NCD conditions. However, the trend of NCD in Malaysia continues to increase throughout the year.

Based on the National Health and Morbidity Survey (NHMS) in Malaysia, the prevalence of diabetes, hypertension, hypercholesterolemia, overweight, and obesity remains high among the population [1]. Moreover, NHMS from 2015 to 2019 reported that there is an increasing trend of NCDs such as diabetes and hypertension among the low-income group compared to other income groups [2.3]. This demonstrates an increasing trend of NCD among the population, thereby increasing their risk of NCD.

The rise of NCDs significantly impacts individuals' health, Quality of Life (QoL), and workforce productivity, causing increased absenteeism, decreased work performance, and a financial burden on the country [4]. Indeed, the underprivileged are unable to access good health care and facilities, putting them at high risk for NCD [5]. Moreover, they also lose their source of family income to cover the cost of treatment and healthcare, or to bear the financial burden of NCD [6]. Thus, it appears that low-income earners had the most significant impact on this issue.

Addressing NCD requires a comprehensive, efficient, and cost-effective approach to improve health and well-being. Additionally, understanding the factors associated with NCD is crucial for developing health interventions for this disadvantaged group. NCD management is one approach to improve the QoL and prevent complications of the disease. It is also considered an

effective and low-cost initiative for preventing and managing NCDs [7,8]. This is consistent with a previous study, which reported a significant relationship between QoL, general health, and management of NCD [9].Therefore, NCD management is one of the best approaches to improving the QoL and well-being.

Broadly, NCD is a complex condition that requires engagement from multiple contributors, including individual, social, community, and societal factors [10]. This is mainly attributed to the fact that personal, social, organizational, community, and societal factors can positively or negatively impact an individual's influence on behavior. Therefore, effective strategies for NCD management require multi-sectoral collaboration and action. This is aligned with the National Strategic Plan for Non-Communicable Diseases (NSP-NCD) 2016–2025, which provides a comprehensive framework to strengthen strategies for the prevention and control of non-communicable diseases in Malaysia, with an emphasis on a whole-of-government and whole-of-society approach.

In this study, Socio-Ecological Model (SEM) is one of the most commonly utilized theories formulated by McLeroy et al. (1988) to explain environmental causes of health behavior and health promotion. This includes identifying interventions that offer five levels of influence specific to health behavior, such as intrapersonal, interpersonal, organizational, community, and societal factors [11]. In addition, the SEM model can provide a comprehensive understanding of the various influences on health outcomes and on reducing health inequality, especially among low-income groups [12].

In line with this, attitude plays a crucial role in shaping human behavior and can be considered a mediator of preventive health behaviors. Attitude is the evaluation, feelings, and tendencies towards objects, events, groups, or symbols that significantly influence individuals to engage in actions that promote health and prevent disease. Attitude can also

drive motivation to initiate health-related behavioral actions. Essentially, individuals who have a positive attitude can influence engagement to behave towards the management of NCDs. In contrast, a negative attitude implies a low attitude evaluation that can influence and hinder the development of good NCD management. This suggests that attitude is a psychological factor in shaping decisions that encourage a person to engage in preventive behavior. Therefore, an important emphasis on understanding predictors and mediating attitudes should be studied in determining behavior related to NCD management, as the nature of this disease is characterized by a long duration, slow progression, incurability, and lifelong conditions. In other words, continuous management of NCDs needs to be emphasized. Nevertheless, there remains a limited number of studies on the management of NCDs among low-income households. Most studies on NCD among low-income earners have more focus on several factors such as demographic, socio-economic and health characteristics [6], prevalence of NCDs, socio-demographic and socio-economic factors [13,14], socio-demographic factors [15], demographic and socio-economic factors [16], health status and living needs [17], health, mental health, health and nutritional behaviors [18], and QoL [19]. Furthermore, there is still a lack of local researchers who utilize the SEM to assess the effect of attitude as a mediator in assessing socio-ecological factors (e.g., intrapersonal, interpersonal, organizational, community, and societal) and NCD management. Building on this, the role of attitude as a mediator is still limited in studying a relationship [20]. Therefore, this study was conducted to examine the mediating role of attitude on the relationship between socio-ecological factors and NCD management among low-income households in Malaysia.

Material and Methods

Study Design and Study Location

A cross-sectional study was conducted among support staff at selected ministries in Putrajaya. Putrajaya is located approximately 25 kilometers south of the capital, Kuala Lumpur. It is the administrative center of the Malaysian government, replacing Kuala Lumpur as the Government Administrative Center to avoid congestion around the Kuala Lumpur area. Putrajaya is a planned city that features ministries, housing for civil servants, lakes, and green areas, designed to provide peace from the stress of modern life. Thus, this location was selected, as it is the Federal Government Administration Center, which represents the largest number of civil servants in Malaysia.

Sampling Technique and Sample

A cluster random sampling technique was employed in this study. Of 24 ministries, eight ministries were randomly selected to represent the ministries in Putrajaya. Subsequently, the respondents were selected using random sampling based on the criteria: permanent support staff aged between 18 and 60 years old and having NCDs. Meanwhile, exclusion criteria were contract or part-time staff and those who refused to participate.

Sample Size

Sample size based on an online calculator for Structural Equation Modeling (SEM) of Soper, D.S. (2020) by setting the anticipated effect size $f^2 = 0.3$, the desired p-value was at 0.05, the desired statistical power level at 80%, the number of observed variables was 94, the number of latent variables was 12, and the number of minimum sample sizes suggested is 200 [21]. According to Kline (2010), the sample size for SEM should be in the range of 100 to 200 subjects, which is considered a medium-sized sample, while a sample size above 200 is

considered large. Hence, this study considered 200 samples, adjusted for a 20% non-response rate, and the minimum sample size required was 240 [22].

Instruments

A set of questionnaires was distributed to respondents who fulfilled the inclusion criteria via an online survey using a Google Form link. This study instrument comprises four sections, which include socio-demographic information, knowledge of NCDs, attitudes towards NCDs, and the Chronic Illness Resources Survey (CIRS). The questionnaire was adapted from previous studies [23,24]. Section A consists of socio-demographic information, including age, gender, race, marital status, educational level, and monthly household income, smoking status, Body Mass Index (BMI) status, and taking medications status. Section B assesses the respondents' knowledge of NCD. The knowledge questions cover the understanding of NCD, including the major NCD such as cardiovascular disease, stroke, and hypertension, diabetes mellitus, and chronic obstructive pulmonary disease, as well as risk factors, complications, and disease management. Respondents were instructed to respond with 'yes,' 'no,' and 'do not know.' Knowledge levels were classified as poor (0-50%), moderate (51-75%), and good (76-100%). It had excellent internal consistency reliability (Cronbach's α = 0.961). Section C consists of 15 items assessing attitudes towards NCD, including balanced diet, physical activity, smoking, salt intake, medication intake, and treatment checks. The question was evaluated using a 5-point Likert scale ranging from 'Strongly Agree,' 'Agree,' 'Not Sure', 'Disagree,' and 'Strongly Disagree'. The total score was divided into three categories as follows: poor attitude (scores 0-50%), moderate attitude (scores 51-75%), and good attitude (scores 76-100%). In this study, the survey revealed good internal consistency reliability (Cronbach's α= 0.898). Section D was the final section of the questionnaire containing 65 items for the CIRS to assess socio-ecological factors, divided into five sub-scales that measure

interpersonal, organizational, community, societal, and disease management. The questionnaire is based on a 5-point Likert scale: ‘Not at all,’ ‘A little,’ ‘Moderate,’ ‘Quite a lot,’ and ‘A great deal.’ The total score can be categorized as follows: less than 60% is considered low, 60% to 80% is considered moderate, and 80% and above is considered high. The Cronbach’s Alpha for the dimensions of the questionnaire ranged from 0.70 to 0.90, demonstrating good reliability.

Ethics considerations

Ethical approval for this study was obtained from the Ethics Committee for Research Involving Human Subjects, Universiti Putra Malaysia (Reference: JKEUPM-2024-301). Prior to data collection, all respondents were provided with written informed consent that explained their participation in the study was voluntary and outlined the study’s purpose, risks, and benefits. The confidentiality of the information was maintained in the strictest confidence, and no names or identifying information were recorded to ensure participant anonymity during data analysis.

Data Analysis

Data analysis was performed using the Statistical Package of Social Sciences (SPSS) version 27 and the Analysis of Moment Structure (AMOS) version 24. Meanwhile, descriptive analysis was performed using frequencies and percentages. Following this, SEM analysis was employed to examine the interrelationship between the constructs in the hypothesized model. Subsequently, a test of the mediation effect was performed through a bootstrap analysis. The significance level was set at $p < 0.05$.

Results

Socio-demographic characteristics

The socio-demographic characteristics of the respondents are summarized in Table 1. The majority of the respondents were aged between 40 and 49 years old (67.5%), female (69.5%), Malay (95.0%), and married (73.5%). The household income of the respondents ranges between RM3501 and RM4000 (51.0%), and they they received a higher educational level of at STPM/ Matriculation/ Diploma and SPM/Certificate (69.0%). Approximately 43.0% of the respondents had high cholesterol, while 41.0% of them were obese. The majority of them (93.0%) were non-smokers. More than half of them (56.0%) were taking medications regularly.

Table 1. Socio-demographic characteristics of the respondents

Socio-demographic Characteristics	Frequency (n)	Percentage (%)
**Age**		
Below than 30 years old	11	5.5
30 - 39 years old	43	21.5
40 - 49 years old	135	67.5
50 - 59 years old	11	5.5
**Gender**		
Male	61	30.5
Female	139	69.5
**Ethnicity**		
Malay	190	95.0
Chinese	7	3.5
Indian	0	0.0
Others	3	1.5
**Marital Status**		
Single	29	14.5
Married	147	73.5

Widowed/Divorced/Separated	24	12.0
**Monthly Household Income**		
RM1001 - RM1500	0	0.0
RM1501 - RM2000	18	9.0
RM2001 - RM2500	16	8.0
RM2501 - RM3000	21	10.5
RM3001 - RM3500	37	18.5
RM3501 - RM4000	102	51.0
More than RM4000	6	3.0
**Educational Level**		
Degree	62	31.0
STPM/ Matriculation/ Diploma	69	34.5
SPM/ Certificate	69	34.5
PT3/ PMR/ SRP	0	0.0
**Types of Non-Communicable Disease**		
Diabetes Mellitus	55	27.5
Heart Disease	10	5.0
Stroke	3	1.5
Chronic Obstructive Pulmonary Disease (COPD)	4	2.0
High Cholesterol	86	43.0
Cancer	3	1.5
Kidney Disease	4	2.0
**Body Mass Index (BMI)**		
Underweight	11	5.5
Normal	38	19.0
Overweight	69	34.5
Obese	82	41.0
**Smoking status**		
Yes	14	7.0
No	186	93.0
**Status of prescribed medication intake**		
Yes	112	56.0

No	88	44.0

Structural Equation Modeling Analysis

Structural Equation Modeling (SEM) was employed to assess the hypotheses of the study. Using this method, hypotheses can explain relationships among multiple variables and the degree of their correlations with the data obtained simultaneously. Subsequently, the model fit was determined based on the Goodness-of-Fit (GOF) [25]. GOF is based on how well the data predicted by the model correspond to the data that were actually collected. The closer the values of these two matrices are to each other, the better the model is considered to fit. GOF indices should be reported include $\chi 2$ value, relative ($\chi 2$/df), absolute fit indices (i.e., Goodness-of-Fit Index (GFI), and Root Mean Square Error of Approximation (RMSEA), and incremental fit indices (i.e., Comparative Fit Index (CFI), Normed Fit Index (NFI), and Tucker-Lewis Index (TLI)). Based on the GOF indices, the model is considered fit when the values of the GFI and the Adjusted Goodness-of-Fit Index (AGFI) range between 0 and 1. However, values greater than 0.9 indicate a better and more meaningful model fit. Furthermore, the incremental fit indices such as the CFI, NFI, Incremental Fit Index (IFI), and TLI also suggest a good model fit when their values are approximately 0.9 or higher, with a value of 1 representing a perfect fit to the model. RMSEA is another important measure, where values greater than .1 indicate a poor model fit, while values ranging from .08 to .1 indicate a better fit. Moreover, values ranging from .03 and .08 indicate a better fit model, whereas Chisq/df lower than the upper limit should be from 1.0 to 3.0, while the maximum acceptable limit is 5.0.

Hair et al. (2010) recommended that three to four indices, including one absolute index and one incremental index, meet the criteria, which provides adequate evidence of model fit [25]. Based on SEM, the resulting structural model fits indicated the data fit the model with

[ChiSq/df= 1.693, P = 0.000, CFI = 0.920, TLI = 0.912, RMSEA = 0.059]. The value of the relative chi-square ($\chi 2$/df) was well below 5, indicating an acceptable fit between the proposed measurement model and the collected data. Other fit indices, namely CFI and TLI values, were above the minimum value of .90, and the RMSEA value was below the cut-off value of .08 (RMSEA = .050). Therefore, the analysis of the SEM indicated that the overall value of the fit index reached acceptable levels and confirmed that the structural model was a good fit.

Structural model

In this study, structural model analysis was employed to conduct path analysis and evaluate the research hypotheses. Table 2 summarizes the results of the hypothesized path analysis of the structural model of this study. The influence of intrapersonal factors (knowledge), interpersonal factors (family and friends), organizational factors (workplace psychosocial support), community factors (neighborhood, local community, community organization, and healthcare services), and societal factors (health policy of healthcare financing and mass media) on NCD management was assessed through path analysis. Attitude (β = 1.293, p = 0.000), community factors (β = 0.263, p = 0.000), and societal factors (β = 0.459, p = 0.000) had a significant effect on NCD management. This indicated that community factors, societal factors, and attitude significantly influence NCD management.

Table 2. Regression Weights in Direct Hypothesis Model

Construct	Path	Construct	Estimate	S.E.	C.R.	P
Non-Communicable Disease Management	<---	Attitude	1.293	0.328	3.936	0.000***
Non-Communicable Disease Management	<---	Intrapersonal Factor	-0.020	0.016	-1.298	0.194
Non-Communicable Disease Management	<---	Interpersonal Factors	0.179	0.141	1.269	0.204
Non-Communicable Disease Management	<---	Organizational Factors	0.028	0.146	0.191	0.849

Non-Communicable Disease Management	<---	Community Factors	-0.263	0.060	-4.375	0.000***
Non-Communicable Disease Management	<---	Societal Factors	0.459	0.082	5.608	0.000***

Note: SE: Standard Error; CR: Critical Ratio for regression weight; P: *** < 0.001

Test of mediation effects

The bootstrapping procedure was conducted to evaluate mediation effects. Table 3 outlines the results of attitude mediating the relationship between intrapersonal, interpersonal, organizational, community, and societal factors with NCD management. These findings revealed that the indirect effect of intrapersonal, community, and societal factors on NCD management is mediated by attitude. In explaining the mediation effect, the findings reveal significant effects were observed for community factors in the direct model (Beta = -0.272, p = 0.002) and the indirect model (Beta = -0.100, p = 0.010). Likewise, there were statistically significant effects of societal factors, both in the direct model (Beta = 0.433, p = 0.003) and in the indirect model (Beta = 0.121, p = 0.012). Therefore, it can be concluded that attitude partially mediates the relationship between community and societal factors in NCD management. Nevertheless, the direct effect of the intrapersonal factor (knowledge) on NCD management was not statistically significant (β = -0.068, p = 0.179). When attitude was included as a mediator, the results revealed that the intrapersonal factor (knowledge) on NCD management was significant (β = -0.057, p =0.005). Thus, these findings indicated that attitude fully mediated the relationship between the intrapersonal factor (knowledge) and NCD management.

Table 3. Results of bootstrap analysis

Model/Hypothesized Path	Beta	P	95% CI BC Lower Bound	95% CI BC Upper Bound	Result
**Direct Model:**					
Intrapersonal Factor → Non-Communicable Disease Management	0.068	0.179	-0.168	0.017	Not Significant
**Indirect Model:**					
Intrapersonal Factor → Attitude → Non-Communicable Disease Management	-0.057	0.005	-0.138	-0.024	Significant
**Direct Model:**					
Interpersonal Factors → Non-Communicable Disease Management	0.142	0.283	-0.062	0.389	Not Significant
**Indirect Model:**					
Interpersonal Factors → Attitude → Non-Communicable Disease Management	0.057	0.108	-0.001	0.204	Not Significant

**Direct Model:** Organizational Factor → Non-Communicable Disease Management	0.021	0.833	-0.195	0.198	Not Significant
**Indirect Model:** Organizational Factor → Attitude → Non-Communicable Disease Management	0.005	0.756	-0.064	0.075	Not Significant
**Direct Model:** Community Factors → Non-Communicable Disease Management	-0.272	0.002	-0.394	-0.158	Significant
**Indirect Model:** Community Factors → Attitude → Non-Communicable Disease Management	-0.100	0.010	-0.214	-0.031	Significant
**Direct Model:** Societal Factors → Non-Communicable Disease Management	0.433	0.003	0.246	0.646	Significant
**Indirect Model:** Societal Factors → Attitude → Non-Communicable Disease Management	0.121	0.012	0.032	0.272	Significant

Discussion

This study examines the mediating role of attitude on the relationship between socio-ecological factors and NCD management. This result showed that attitude, community, and social factors influence NCD management. Attitude exerted the greatest direct influence on NCD management. This may be due to having a good attitude; individuals are confident in practice towards the management of chronic disease [26]. Therefore, when individuals have a positive attitude, it helps to improve their management of NCDs. Additionally, findings revealed that community factors (neighborhood, local community, community organization, and healthcare services) influence NCD management. It indicated that community factors effectively influence NCD management. One probable explanation is that the approach of health promotion programs applied in the community has increased their awareness of NCD. This result was consistent with previous studies, which have reported that community-based programs, such as Komuniti Sihat Perkasa Negara (KOSPEN), help address NCDs and prevent and control NCD risk factors [27]. Another study discovered that community engagement involves partnerships with local health organizations, such as community outreach programs and hosting health awareness events, which contribute to the overall well-being of the communities [28]. Therefore, the involvement of community health workers as part of the healthcare team is one of the effective efforts of the community program in increasing access to treatment and healthcare. This indicated that the community approach initiative helps ongoing healthcare by preventing and controlling NCD risk within the community [29]. Another possible reason is that Putrajaya was designed with a neighborhood concept in mind, adopting a garden city concept that provides residents with neighborhood parks, including green

spaces, gardens, and lakes, promoting physical activity (e.g., walking, jogging, and cycling). A previous study noted that open spaces in Putrajaya significantly contribute to health promotion [30]. A similar finding was also noted, highlighting that the presence of green urban spaces promotes healthy communities and healthy lifestyles [31]. Thus, it encourages a healthier lifestyle through community engagement in outdoor activities, fostering community bonds in shared recreational spaces. Furthermore, since the respondents live in an urban area, the majority of them have easy access to healthcare services. Urban areas make it easier for people to access healthcare services due to better public transportation options and a variety of clinic and hospital services, which influence the management of NCDs. In fact, individuals from relatively low-income earners prefer to choose services from their community, where they need only pay MYR 1 for a general outpatient consultation and MYR 5 for a specialist consultation [32]. This is consistent with the report by NHMS 2019, which revealed that 8.1% of B40 households in Malaysia received healthcare services as outpatients in the two weeks prior to consulting healthcare professionals [2]. Moreover, the government's role at the primary care level in NCD management has seen an increase in the roles of doctors, nurses, and medical assistants, where follow-up care is provided at many primary health clinics [33]. This is supported by [34], who mentioned that management in the primary care system provides services to the community for effective disease management and patient empowerment. This subsequently leads to better health status among NCD patients as well as the general community. Furthermore, healthcare services help prevent and treat health problems for the public, which in turn influences the population's overall health status. Therefore, community factors such as access to healthcare facilities, availability of recreational spaces, and community health programs had an effect on NCD management among support staff.

This study also revealed that societal factors (health policy of healthcare financing, and mass media) were identified as significant factors that significantly influenced NCD management. This could be a health policy in healthcare financing by the government, helping to protect them from NCDs. This aligns with the findings of the NHMS (2009-2023), which reported that government spending on health, particularly for low-income groups, reflects a strong public role in health financing for these groups. For example, PeKa B40 aims to improve access to healthcare services for low-income groups [35]. In addition, heavily subsidized by the government, providing affordable and accessible care to all citizens, especially low-income groups. Therefore, government healthcare financing plays a significant role in protecting populations, especially low-income groups. On the other hand, mass media also play a vital role in influencing NCD management. The existence of mass media provides numerous benefits to the public, making it easier for them to obtain, share, and exchange information, which in turn influences healthy behavior. The findings are consistent with prior research indicating that social media influences preventive behavior by increasing awareness and facilitating information exchange [36]. Another study emphasized that the use of mass media influences health behavior [37]. This implies that NCD management can be improved by enhancing attitude, community, and societal factors.

The current study revealed that attitude mediates the relationship between intrapersonal, community, and societal factors on NCD management. This study found that community and societal factors were partially mediated by attitude. The partial mediating role of attitude was due to community and societal factors having direct and indirect relationships with NCD management through attitudes as mediators. Additionally, attitude fully mediates the relationship between the intrapersonal factor of knowledge and NCD management. This indicated that attitude helps

regulate the relationship between intrapersonal, community, and societal factors in NCD management. In other words, improvements in intrapersonal, community, and societal factors encouraged individuals to adopt better attitudes, which further enhanced NCD management. Furthermore, by increasing an individual's level of awareness, their building practices toward the prevention of NCD can be improved. The findings of this study were consistent with previous studies, which have proven attitude to be a mediator in controlling the relationship between exogenous variables and endogenous variables [38-40]. Hence, attitude serves as a mediator, influencing interpersonal, community, and societal factors in NCD management. Taken together, relevant stakeholders might better consider mediated relationships in a tailored intervention towards achieving NCD management.

Conclusion

In summary, attitude, community, and societal factors have direct effects on NCD management based on the results of the SEM analysis. The mediation test revealed that attitude acts as a partial mediator in the relationship between community and societal factors towards NCD management. Meanwhile, attitude serves as a full mediator in the relationship between intrapersonal factors and NCD management.

To the best of our knowledge, this is the first study to investigate the mediating effects of attitude on the relationship between socio-ecological factors and NCD management. This study presents a new approach by integrating socio-ecological factors through attitudes as a mediator in NCD management. The research also provides new insights for relevant stakeholders, including policymakers, healthcare

providers, and programme managers, by offering a better understanding of these influences and the socio-ecological approach to designing holistic interventions for the future. However, the study has limitations, such as the use of self-report questionnaires, which may have given false or incomplete responses. Additionally, the cross-sectional design makes it impossible to establish causality, which longitudinal studies could help determine. In addition, this study only utilized socio-ecological factors (e.g., intrapersonal, interpersonal, organizational, community, and societal). The assessment of other factors, such as health literacy and self-efficacy, should be considered in this study, as they can contribute to a deeper understanding of NCD management. Lastly, future studies can also be conducted on larger samples by utilizing SEM analysis to confirm the good results and generalizability.

References

1. Institute of Public Health, Technical Report National Health and Morbidity Survey (NMHS) 2023. Non-Communicable Disease and Healthcare Demand, Malaysia, 2024.

2. Institute of Public Health, National Health and Morbidity Survey 2019 (NHMS 2019). Non-communicable diseases, healthcare demand, and health literacy. Kuala Lumpur: Ministry of Health Malaysia, 2019.

3. Institute of Public Health, National Health and Morbidity Survey 2015 (NHMS 2015). Vol. II: Non-Communicable Diseases, Risk Factors & Other Health Problems. Kuala Lumpur: Ministry of Health Malaysia, 2015.

4. Budreviciute A, Damiati S, Sabir DK, Onder K, Schuller-Goetzburg P, Plakys G, , et al. Management and prevention strategies for non-communicable diseases (NCDs) and their risk factors. Front. public health. 2020; 8:574111. https://doi: 10.3389/fpubh.2020.574111.

5. Stringhini S, Carmeli C, Jokela M, Avendaño M, Muennig P, Guida F,et al., Socioeconomic status and the 25× 25 risk factors as determinants of premature mortality: a multicohort study and meta-analysis of 1· 7 million men and women. Lancet. 2017; 389 (10075):1229–1237. https://doi.org/10.1016/S0140-6736(16)32380-7.

6. Isamail N, Said R, Ismail NW, Haron SA. Non-Communicable Diseases Impact Low-Income Households in Malaysia. Malaysian Journal of Medical Sciences. 2024;31(1): 124. https://doi: 10.21315/mjms2024.31.1.11.

7. PhillipsCM, Chen LW, Heude B, Bernard JY, Harvey NC, Duijts L, et al. Dietary inflammatory index and non-communicable disease risk: a narrative review. Nutrients. 2019;11(8): 1873. https://doi: 10.3390/nu11081873.

8. Lee IM, Shiroma EJ, Lobelo F, Puska P, Blair SN, Katzmarzyk PT. Effect of physical inactivity on major non-communicable diseases worldwide: an analysis of burden of disease and life expectancy. Lancet. 2012;380 (9838): 219 –229. https://doi: 10.1016/S0140-6736(12)61031-9.

9. Lorber M, Reljić NM, Kegl B, Fekonja Z, Štiglic G, Davey A, et al. Person-Centred Care: A Support Strategy for Managing Non-Communicable Diseases. Healthcare. (2024);12(5): 526. https://doi.org/10.3390/healthcare12050526.

10. Nguyen KH, Fields JD, Cemballi AG, Desai R, Gopalan A, Cruz T, et al. The role of community-based organizations in improving chronic care for safety-net populations. Journal of the

American Board of Family Medicine. 2021;34 (4):698-708, https://doi: 10.3122/jabfm.2021.04.200591.

11. McLeroy KR, Bibeau D, Steckler A, Glanz K. An Ecological Perspective on Health Promotion Programs. Health Educ. Q. 1988; 315(4):51–377. https://doi: 10.1177/109019818801500401.
12. Baron SL, Beard S, Davis LK, Delp L, Forst L, Kidd-Taylor A,, et al. Promoting Integrated Approaches to Reducing Health Inequities Among Low- Income Workers: Applying a Social Ecological Framework. Am. J. Ind. Med. 2014;57: 539–556. https://doi: 10.1002/ajim.22174.
13. Hami R, Shahabudin SA, Shariff NM, Mohd Mujar NM, Isa S, Roslan H, et al. Factors Contributing to Non-Communicable Diseases in a Selected Low Socio-Economic Status Community in Kedah, Malaysia. Malaysian Journal of Medicine & Health Sciences. 2021;17(1): 117–123.
14. Harris H, Ooi YB, Lee JS, Matanjun P. Non-communicable diseases among low income adults in rural coastal communities in Eastern Sabah, Malaysia. BMC Public Health. 2019;19: 1–13. https://doi.org/10.1186/s12889-019-6854-6.
15. Fiidow OA, Huda BZ, Salmiah MS. Salmiah, Socio-demographic factors of hypertension among non-academic staff in Universiti Putra Malaysia. International Journal of Public Health and Clinical Sciences. 2016;3(4):158 –168.
16. SuTT, Amiri M, Mohd Hairi F, Thangiah N, Bulgiba A, Majid HA. Prediction of Cardiovascular Disease Risk among Low-Income Urban Dwellers in Metropolitan Kuala Lumpur, Malaysia. BioMed Res Int. 2015;2015(1): 516984. https:// doi: 10.1155/2015/516984.
17. Puteh SE, Kadir HA.. Factors associated with health status and living needs impact on b40 women's quality of life in Malaysia: an analysis based on b40 rural and urban poor areas.

Malaysian J. Public Health Med. 2022; 22(2):176–186. https://doi.org/10.37268/mjphm/vol.22/no.2/art.1717.

18. Shahar S, Lau H, Puteh SE, Amara S, Razak NA. Health, access and nutritional issues among low-income population in Malaysia: Introductory note. BMC Public Health. 2019;19: 1–5. https://doi.org/10.1186/s12889-019-6852-8.
19. Wan PutehSE, Siwar C, Zaidi MA, Abdul Kadir H. Health related quality of life (HRQOL) among low socioeconomic population in Malaysia. BMC Public Health. 2019;19: 1–8. https://doi.org/10.1186/s12889- 019-6853-7.
20. ÇabukS, Tanrikulu C, Gelibolu L. Understanding organic food consumption: attitude as a mediator. International Journal of Consumer Studies. 2014;38: 337–345. https://doi.org/10.1111/ijcs.12094.
21. Soper DS, A-Priori Sample Size Calculator for Structural Equation Models [Software]. http://wwwdanielsopercom/statcalc, 2020.
22. Kline RB. Principles and practice of Structural Equation Modelling (3rd ed). New York: The Guilford Press. 2010.
23. Ithnin M, Nor NA, Nordin NJ, Effendy NM, Sahar MA, Abdullah KH,, et al. Knowledge, attitude, and practice on Non- Communicable Diseases (NCDs) among the adult population in the urban area of Negeri Sembilan, Malaysia. International Journal of Research in Pharmaceutical Sciences. 2018; 9 (2Suppl): 88–94. https://doi.org/10.1080/14635240.2020.1749526.
24. Glasgow RE, Strycker LA, Toobert DJ, Eakin E. A Social-Ecologic Approach to Assessing Support for Disease Self- Management: The Chronic Illness Resources Surveys. J. Behav. Med. 2000;23(6): 559-583. https:// doi:.10.1023/a:1005507603901.

25. Hair F, Black WC, Anderson RE. Multivariate Data Analysis. (7th Ed). New York: Pearson. 2010.
26. Ithnin M, Nor NA, Juliana N, Effendy NM, Sahar MA, Abdullah KH, et al. Knowledge, Attitude and Practices Towards Lifestyle Related Non-Communicable Diseases (NCDs): A Cross Sectional Study among Indigenous Orang Asli Adults in Negeri Sembilan, Malaysia. IIUM Medical Journal Malaysia. 2020;19 (2):75–82. https://doi.org/10.31436/imjm.v19i2.1569.
27. Lim KH, M Fadhli Y, Omar M, Rosnah R, M. Nazaruddin B, Sumarni MG, et al. Evulation of Effectiveness of Implementation of "Komuniti Sihat Perkasa Negara (KOSPEN) Programme in Malaysia- Phase 1. Kuala Lumpur: Ministry of Health Malaysia, 2015.
28. Kabuya KE. Influence of Workplace Health on Awareness of Non-Communicable Diseases (NCDs) and Its Cascading Effects on the Well-being of Families and Communities. International Journal of Sciences: Basic and Applied Research. 2024. 73 (1):43–58.
29. Kataria I, Ngongo C, Lim SC, Kocher E, Kowal P, Chandran A, et al. Development and evaluation of a digital, community-based intervention to reduce noncommunicable disease risk in a low-resource urban setting in Malaysia: a research protocol. Implementation science communications. 2020;1: 1–9. https://doi.org/10.1186/s43058-020-00080-y.
30. Danjaji AS, Danladi M, Adamu A, Danladi HM. Urban Green Spaces as Promoters of Healthy Living: Evidence from Putrajaya, Malaysia. Research Square. 2021: 1 –17. https://doi.org/10.21203/rs.3.rs-1166766/v1.
31. Ayob Z, A.R. Amir AR. Developing Urban Walkability Measures Grounded in Green Urbanism Principles Using The Delphi Survey Study. Planning Malaysia. 2024; 22:307–320.
32. Aizuddin AN, Al Junid SM. Willingness to pay for outpatient services user fees: Malaysian community perspective. Jurnal Sains Kesihatan Malaysia. 2018; 16(1):145-153.

33. Mustapha FI, Omar ZA, Mihat O, Md Noh K, Hassan N, Abu Bakar R, et al. Addressing non-communicable diseases in Malaysia: an integrative process of systems and community. BMC Public Health. 2014;14: 1–6. htttps://doi:doi: 10.1186/1471-2458-14-S2-S4.

34. Fadzilah A, Azima R, Khalsom S, Rosliza AM. Governance framework in non-communicable disease (NCD) control and prevention programme at primary care level in Malaysia. International Journal of Public Health and Clinical Sciences. (2020); 7(3):167-185.

35. Yunus SZ, Puteh SE, Ali AM, Daud F., S.E.W. Puteh, A.M. Ali, F. Daud, The Covid impact to public healthcare utilization among urban low-income subsidized community in Klang Valley Malaysia. Health Serv. Res. Manag, Epidemiol. 2021;8: 1–9. https://doi: 10.1177/23333928211002407.

36. Nazir M, Hussain I, Tian J, Akram S, Mangenda Tshiaba S, Mushtaq S, et al. A multidimensional model of public health approaches against COVID-19. International Journal of Environmental Research and Public Health. 2020; 17(11): (3780. https://doi.org/10.3390/ijerph17113780.

37. Nabi RL, Huskey R, Nicholls SB, Keblusek L, Reed M. When audiences become advocates: Self-induced behavior change through health message posting in social media. Computers in Human Behavior. 2019;99: 260–267. https://doi.org/10.1016/j.chb.2019.05.030.

38. Zhang Y, Zhang B, Chen C, Feng X, Song S, Wang H. The mediation effect of attitude on the association between knowledge and self-management behaviors in Chinese patients with diabetes. International Journal of Public Health. 2023; 68: 1606022. https;//doi: 10.3389/ijph.2023.1606022.

39. Saengcha S, Pattanapongthorn J, Jermsittiparsert K. Influence of Beliefs on Attitudes and Intention of Adopting e-Health System among the Public Hospitals of Thailand. International Journal of Innovation, Creativity and Change. 2019; 8(8):238–255.

40. An HG, Ha JY, Kim SH. Mediating Effect of Attitude on the Relationship between Knowledge about Tuberculosis and Preventive Behavior Toward Tuberculosis of Foreign Students. International Journal of Bio-Science and Bio-Technology. 2016;8(2):181–196. https://dx.doi.org/10.14257/ijbsbt.2016.8.2.17.

Click here to access/download
Supporting Information
Questionnaire.docx

Click here to access/download
Supporting Information
Instrument.docx

TYPE OF MANUSCRIPT: ORIGINAL RESEARCH ARTICLE

Mediating Role of Attitude on the Relationship Between Socio-Ecological Factors and Non-Communicable Disease Management among Support Staff in Putrajaya, Malaysia

Azrin Shah Abu Bakar[1], Haliza Abdul Rahman[1,2*], Ahmad Iqmer Nashriq Mohd Nazan[3]

[1] *Department of Environmental and Occupational Health, Faculty of Medicine and Health Sciences, Universiti Putra Malaysia, 43400 Serdang, Selangor, Malaysia.*
[2] *Institute for Social Science Studies, Universiti Putra Malaysia, 43400 Serdang, Selangor, Malaysia.*
[3]*Department of Community Health, Faculty of Medicine and Health Sciences, Universiti Putra Malaysia, 43400 Serdang, Selangor, Malaysia.*

***** dr.haliza@upm.edu.my

Mediating Role of Attitude on the Relationship Between Socio-Ecological Factors and Non-Communicable Disease Management among Support Staff in Putrajaya, Malaysia

ABSTRACT

Non-Communicable Disease (NCD) is a major global health issue that contributes to morbidity and mortality problems across countries including Malaysia. Social environment is increasingly recognized as critical factors in shaping the development of NCDs that affect particularly vulnerable groups, especially low-income households, in managing chronic diseases. However, there was still limited information regarding the relationship between socio-ecological factors (i.e., intrapersonal, interpersonal, organizational, community, and societal factors) and NCD management through attitude. Therefore, this study aimed to examine the mediating role of attitude on the relationship between socio-ecological factors and NCD management. This study employs a cross-sectional survey of 200 support staff with NCD at selected ministries in Putrajaya, Malaysia. The data collected included socio-demographic information, knowledge of NCDs, attitudes towards NCDs, and the Chronic Illness Resources Survey (CIRS). Statistical Package of Social Sciences (SPSS) version 27 and Analysis of Moment Structures (AMOS) version 24 were employed to analyze the data through descriptive analysis and Structural Equation Model (SEM). The path analysis revealed that attitude, community, and societal factors affect NCD management. Further, mediation analysis indicated that attitude mediates the relationship between intrapersonal, community, and societal factors and NCD management. This study suggests that relevant stakeholders, including policymakers, healthcare providers, and program managers should pay attention to intrapersonal, community, and societal factors. This can be achieved through the implementation of strategies, policies, and planning, as well as designing holistic interventions to improve NCD management.

Keywords: Attitude, Socio-ecological factors, Non-communicable Disease Management, Support staff, Putrajaya, Malaysia

Introduction

Globalization, urbanization, technological advancements, and demographic (population aging) have contributed significantly to the increase in Non-Communicable Diseases (NCDs) in Malaysia. Despite various efforts undertaken by the government including programs, activities, campaigns, health screenings, and community participation in health promotion focus on managing NCD conditions. However, the trend of NCD in Malaysia continues to increase throughout the year.

Based on the National Health and Morbidity Survey (NHMS) in Malaysia, the prevalence of diabetes, hypertension, hypercholesterolemia, overweight, and obesity remains high among the population [1]. Moreover, NHMS from 2015 to 2019 reported that there is an increasing trend of NCDs such as diabetes and hypertension among the low-income group compared to other income groups [2.3]. This demonstrates an increasing trend of NCD among the population, thereby increasing their risk of NCD.

The rise of NCDs significantly impacts individuals' health, Quality of Life (QoL), and workforce productivity, causing increased absenteeism, decreased work performance, and a financial burden on the country [4]. Indeed, the underprivileged are unable to access good health care and facilities, putting them at high risk for NCD [5]. Moreover, they also lose their source of family income to cover the cost of treatment and healthcare, or to bear the financial burden of NCD [6]. Thus, it appears that low-income earners had the most significant impact on this issue.

Addressing NCD requires a comprehensive, efficient, and cost-effective approach to improve health and well-being. Additionally, understanding the factors associated with NCD is crucial for developing health interventions for this disadvantaged group. NCD management is one approach to improve the QoL and prevent complications of the disease. It is also considered an

effective and low-cost initiative for preventing and managing NCDs [7,8]. This is consistent with a previous study, which reported a significant relationship between QoL, general health, and management of NCD [9].Therefore, NCD management is one of the best approaches to improving the QoL and well-being.

Broadly, NCD is a complex condition that requires engagement from multiple contributors, including individual, social, community, and societal factors [10]. This is mainly attributed to the fact that personal, social, organizational, community, and societal factors can positively or negatively impact an individual's influence on behavior. Therefore, effective strategies for NCD management require multi-sectoral collaboration and action. This is aligned with the National Strategic Plan for Non-Communicable Diseases (NSP-NCD) 2016–2025, which provides a comprehensive framework to strengthen strategies for the prevention and control of non-communicable diseases in Malaysia, with an emphasis on a whole-of-government and whole-of-society approach.

In this study, Socio-Ecological Model (SEM) is one of the most commonly utilized theories formulated by McLeroy et al. (1988) to explain environmental causes of health behavior and health promotion. This includes identifying interventions that offer five levels of influence specific to health behavior, such as intrapersonal, interpersonal, organizational, community, and societal factors [11]. In addition, the SEM model can provide a comprehensive understanding of the various influences on health outcomes and on reducing health inequality, especially among low-income groups [12].

In line with this, attitude plays a crucial role in shaping human behavior and can be considered a mediator of preventive health behaviors. Attitude is the evaluation, feelings, and tendencies towards objects, events, groups, or symbols that significantly influence individuals to engage in actions that promote health and prevent disease. Attitude can also

drive motivation to initiate health-related behavioral actions. Essentially, individuals who have a positive attitude can influence engagement to behave towards the management of NCDs. In contrast, a negative attitude implies a low attitude evaluation that can influence and hinder the development of good NCD management. This suggests that attitude is a psychological factor in shaping decisions that encourage a person to engage in preventive behavior. Therefore, an important emphasis on understanding predictors and mediating attitudes should be studied in determining behavior related to NCD management, as the nature of this disease is characterized by a long duration, slow progression, incurability, and lifelong conditions. In other words, continuous management of NCDs needs to be emphasized. Nevertheless, there remains a limited number of studies on the management of NCDs among low-income households. Most studies on NCD among low-income earners have more focus on several factors such as demographic, socio-economic and health characteristics [6], prevalence of NCDs, socio-demographic and socio-economic factors [13,14], socio-demographic factors [15], demographic and socio-economic factors [16], health status and living needs [17], health, mental health, health and nutritional behaviors [18], and QoL [19]. Furthermore, there is still a lack of local researchers who utilize the SEM to assess the effect of attitude as a mediator in assessing socio-ecological factors (e.g., intrapersonal, interpersonal, organizational, community, and societal) and NCD management. Building on this, the role of attitude as a mediator is still limited in studying a relationship [20]. Therefore, this study was conducted to examine the mediating role of attitude on the relationship between socio-ecological factors and NCD management among low-income households in Malaysia.

Material and Methods

Study Design and Study Location

A cross-sectional study was conducted among support staff at selected ministries in Putrajaya. Putrajaya is located approximately 25 kilometers south of the capital, Kuala Lumpur. It is the administrative center of the Malaysian government, replacing Kuala Lumpur as the Government Administrative Center to avoid congestion around the Kuala Lumpur area. Putrajaya is a planned city that features ministries, housing for civil servants, lakes, and green areas, designed to provide peace from the stress of modern life. Thus, this location was selected, as it is the Federal Government Administration Center, which represents the largest number of civil servants in Malaysia.

Sampling Technique and Sample

A cluster random sampling technique was employed in this study. Of 24 ministries, eight ministries were randomly selected to represent the ministries in Putrajaya. Subsequently, the respondents were selected using random sampling based on the criteria: permanent support staff aged between 18 and 60 years old and having NCDs. Meanwhile, exclusion criteria were contract or part-time staff and those who refused to participate.

Sample Size

Sample size based on an online calculator for Structural Equation Modeling (SEM) of Soper, D.S. (2020) by setting the anticipated effect size $f^2 = 0.3$, the desired p-value was at 0.05, the desired statistical power level at 80%, the number of observed variables was 94, the number of latent variables was 12, and the number of minimum sample sizes suggested is 200 [21]. According to Kline (2010), the sample size for SEM should be in the range of 100 to 200 subjects, which is considered a medium-sized sample, while a sample size above 200 is

considered large. Hence, this study considered 200 samples, adjusted for a 20% non-response rate, and the minimum sample size required was 240 [22].

Instruments

A set of questionnaires was distributed to respondents who fulfilled the inclusion criteria via an online survey using a Google Form link. This study instrument comprises four sections, which include socio-demographic information, knowledge of NCDs, attitudes towards NCDs, and the Chronic Illness Resources Survey (CIRS). The questionnaire was adapted from previous studies [23,24]. Section A consists of socio-demographic information, including age, gender, race, marital status, educational level, and monthly household income, smoking status, Body Mass Index (BMI) status, and taking medications status. Section B assesses the respondents' knowledge of NCD. The knowledge questions cover the understanding of NCD, including the major NCD such as cardiovascular disease, stroke, and hypertension, diabetes mellitus, and chronic obstructive pulmonary disease, as well as risk factors, complications, and disease management. Respondents were instructed to respond with 'yes,' 'no,' and 'do not know.' Knowledge levels were classified as poor (0-50%), moderate (51-75%), and good (76-100%). It had excellent internal consistency reliability (Cronbach's α = 0.961). Section C consists of 15 items assessing attitudes towards NCD, including balanced diet, physical activity, smoking, salt intake, medication intake, and treatment checks. The question was evaluated using a 5-point Likert scale ranging from 'Strongly Agree,' 'Agree,' 'Not Sure', 'Disagree,' and 'Strongly Disagree'. The total score was divided into three categories as follows: poor attitude (scores 0-50%), moderate attitude (scores 51-75%), and good attitude (scores 76-100%). In this study, the survey revealed good internal consistency reliability (Cronbach's α= 0.898). Section D was the final section of the questionnaire containing 65 items for the CIRS to assess socio-ecological factors, divided into five sub-scales that measure

interpersonal, organizational, community, societal, and disease management. The questionnaire is based on a 5-point Likert scale: 'Not at all,' 'A little,' 'Moderate,' 'Quite a lot,' and 'A great deal.' The total score can be categorized as follows: less than 60% is considered low, 60% to 80% is considered moderate, and 80% and above is considered high. The Cronbach's Alpha for the dimensions of the questionnaire ranged from 0.70 to 0.90, demonstrating good reliability.

Ethics considerations

Ethical approval for this study was obtained from the Ethics Committee for Research Involving Human Subjects, Universiti Putra Malaysia (Reference: JKEUPM-2024-301). Prior to data collection, all respondents were provided with written informed consent that explained their participation in the study was voluntary and outlined the study's purpose, risks, and benefits. The confidentiality of the information was maintained in the strictest confidence, and no names or identifying information were recorded to ensure participant anonymity during data analysis.

Data Analysis

Data analysis was performed using the Statistical Package of Social Sciences (SPSS) version 27 and the Analysis of Moment Structure (AMOS) version 24. Meanwhile, descriptive analysis was performed using frequencies and percentages. Following this, SEM analysis was employed to examine the interrelationship between the constructs in the hypothesized model. Subsequently, a test of the mediation effect was performed through a bootstrap analysis. The significance level was set at $p < 0.05$.

Results

Socio-demographic characteristics

The socio-demographic characteristics of the respondents are summarized in Table 1. The majority of the respondents were aged between 40 and 49 years old (67.5%), female (69.5%), Malay (95.0%), and married (73.5%). The household income of the respondents ranges between RM3501 and RM4000 (51.0%), and they they received a higher educational level of at STPM/ Matriculation/ Diploma and SPM/Certificate (69.0%). Approximately 43.0% of the respondents had high cholesterol, while 41.0% of them were obese. The majority of them (93.0%) were non-smokers. More than half of them (56.0%) were taking medications regularly.

Table 1. Socio-demographic characteristics of the respondents

Socio-demographic Characteristics	Frequency (n)	Percentage (%)
**Age**		
Below than 30 years old	11	5.5
30 - 39 years old	43	21.5
40 - 49 years old	135	67.5
50 - 59 years old	11	5.5
**Gender**		
Male	61	30.5
Female	139	69.5
**Ethnicity**		
Malay	190	95.0
Chinese	7	3.5
Indian	0	0.0
Others	3	1.5
**Marital Status**		
Single	29	14.5
Married	147	73.5

Widowed/Divorced/Separated	24	12.0
**Monthly Household Income**		
RM1001 - RM1500	0	0.0
RM1501 - RM2000	18	9.0
RM2001 - RM2500	16	8.0
RM2501 - RM3000	21	10.5
RM3001 - RM3500	37	18.5
RM3501 - RM4000	102	51.0
More than RM4000	6	3.0
**Educational Level**		
Degree	62	31.0
STPM/ Matriculation/ Diploma	69	34.5
SPM/ Certificate	69	34.5
PT3/ PMR/ SRP	0	0.0
**Types of Non-Communicable Disease**		
Diabetes Mellitus	55	27.5
Heart Disease	10	5.0
Stroke	3	1.5
Chronic Obstructive Pulmonary Disease (COPD)	4	2.0
High Cholesterol	86	43.0
Cancer	3	1.5
Kidney Disease	4	2.0
**Body Mass Index (BMI)**		
Underweight	11	5.5
Normal	38	19.0
Overweight	69	34.5
Obese	82	41.0
**Smoking status**		
Yes	14	7.0
No	186	93.0
**Status of prescribed medication intake**		
Yes	112	56.0

No	88	44.0

Structural Equation Modeling Analysis

Structural Equation Modeling (SEM) was employed to assess the hypotheses of the study. Using this method, hypotheses can explain relationships among multiple variables and the degree of their correlations with the data obtained simultaneously. Subsequently, the model fit was determined based on the Goodness-of-Fit (GOF) [25]. GOF is based on how well the data predicted by the model correspond to the data that were actually collected. The closer the values of these two matrices are to each other, the better the model is considered to fit. GOF indices should be reported include χ2 value, relative (χ2/df), absolute fit indices (i.e., Goodness-of-Fit Index (GFI), and Root Mean Square Error of Approximation (RMSEA), and incremental fit indices (i.e., Comparative Fit Index (CFI), Normed Fit Index (NFI), and Tucker-Lewis Index (TLI)). Based on the GOF indices, the model is considered fit when the values of the GFI and the Adjusted Goodness-of-Fit Index (AGFI) range between 0 and 1. However, values greater than 0.9 indicate a better and more meaningful model fit. Furthermore, the incremental fit indices such as the CFI, NFI, Incremental Fit Index (IFI), and TLI also suggest a good model fit when their values are approximately 0.9 or higher, with a value of 1 representing a perfect fit to the model. RMSEA is another important measure, where values greater than .1 indicate a poor model fit, while values ranging from .08 to .1 indicate a better fit. Moreover, values ranging from .03 and .08 indicate a better fit model, whereas Chisq/df lower than the upper limit should be from 1.0 to 3.0, while the maximum acceptable limit is 5.0.

Hair et al. (2010) recommended that three to four indices, including one absolute index and one incremental index, meet the criteria, which provides adequate evidence of model fit [25]. Based on SEM, the resulting structural model fits indicated the data fit the model with

[ChiSq/df= 1.693, P = 0.000, CFI = 0.920, TLI = 0.912, RMSEA = 0.059]. The value of the relative chi-square ($\chi2/df$) was well below 5, indicating an acceptable fit between the proposed measurement model and the collected data. Other fit indices, namely CFI and TLI values, were above the minimum value of .90, and the RMSEA value was below the cut-off value of .08 (RMSEA = .050). Therefore, the analysis of the SEM indicated that the overall value of the fit index reached acceptable levels and confirmed that the structural model was a good fit.

Structural model

In this study, structural model analysis was employed to conduct path analysis and evaluate the research hypotheses. Table 2 summarizes the results of the hypothesized path analysis of the structural model of this study. The influence of intrapersonal factors (knowledge), interpersonal factors (family and friends), organizational factors (workplace psychosocial support), community factors (neighborhood, local community, community organization, and healthcare services), and societal factors (health policy of healthcare financing and mass media) on NCD management was assessed through path analysis. Attitude ($\beta = 1.293$, $p = 0.000$), community factors ($\beta = 0.263$, $p = 0.000$), and societal factors ($\beta = 0.459$, $p = 0.000$) had a significant effect on NCD management. This indicated that community factors, societal factors, and attitude significantly influence NCD management.

Table 2. Regression Weights in Direct Hypothesis Model

Construct	Path	Construct	Estimate	S.E.	C.R.	P
Non-Communicable Disease Management	<---	Attitude	1.293	0.328	3.936	0.000***
Non-Communicable Disease Management	<---	Intrapersonal Factor	-0.020	0.016	-1.298	0.194
Non-Communicable Disease Management	<---	Interpersonal Factors	0.179	0.141	1.269	0.204
Non-Communicable Disease Management	<---	Organizational Factors	0.028	0.146	0.191	0.849

Non-Communicable Disease Management	<---	Community Factors	-0.263	0.060	-4.375	0.000***
Non-Communicable Disease Management	<---	Societal Factors	0.459	0.082	5.608	0.000***

Note: SE: Standard Error; CR: Critical Ratio for regression weight; P: *** < 0.001

Test of mediation effects

The bootstrapping procedure was conducted to evaluate mediation effects. Table 3 outlines the results of attitude mediating the relationship between intrapersonal, interpersonal, organizational, community, and societal factors with NCD management. These findings revealed that the indirect effect of intrapersonal, community, and societal factors on NCD management is mediated by attitude. In explaining the mediation effect, the findings reveal significant effects were observed for community factors in the direct model (Beta = -0.272, p = 0.002) and the indirect model (Beta = -0.100, p = 0.010). Likewise, there were statistically significant effects of societal factors, both in the direct model (Beta = 0.433, p = 0.003) and in the indirect model (Beta = 0.121, p = 0.012). Therefore, it can be concluded that attitude partially mediates the relationship between community and societal factors in NCD management. Nevertheless, the direct effect of the intrapersonal factor (knowledge) on NCD management was not statistically significant (β = -0.068, p = 0.179). When attitude was included as a mediator, the results revealed that the intrapersonal factor (knowledge) on NCD management was significant (β = -0.057, p =0.005). Thus, these findings indicated that attitude fully mediated the relationship between the intrapersonal factor (knowledge) and NCD management.

Table 3. Results of bootstrap analysis

Model/Hypothesized Path	Beta	P	95% CI BC		Result
			Lower Bound	Upper Bound	
**Direct Model:**					
Intrapersonal Factor → Non-Communicable Disease Management	0.068	0.179	-0.168	0.017	Not Significant
**Indirect Model:**					
Intrapersonal Factor → Attitude → Non-Communicable Disease Management	-0.057	0.005	-0.138	-0.024	Significant
**Direct Model:**					
Interpersonal Factors → Non-Communicable Disease Management	0.142	0.283	-0.062	0.389	Not Significant
**Indirect Model:**					
Interpersonal Factors → Attitude → Non-Communicable Disease Management	0.057	0.108	-0.001	0.204	Not Significant

**Direct Model:** Organizational Factor → Non-Communicable Disease Management	0.021	0.833	-0.195	0.198	Not Significant
**Indirect Model:** Organizational Factor → Attitude → Non-Communicable Disease Management	0.005	0.756	-0.064	0.075	Not Significant
**Direct Model:** Community Factors → Non-Communicable Disease Management	-0.272	0.002	-0.394	-0.158	Significant
**Indirect Model:** Community Factors → Attitude → Non-Communicable Disease Management	-0.100	0.010	-0.214	-0.031	Significant
**Direct Model:** Societal Factors → Non-Communicable Disease Management	0.433	0.003	0.246	0.646	Significant
**Indirect Model:** Societal Factors → Attitude → Non-Communicable Disease Management	0.121	0.012	0.032	0.272	Significant

Discussion

This study examines the mediating role of attitude on the relationship between socio-ecological factors and NCD management. This result showed that attitude, community, and social factors influence NCD management. Attitude exerted the greatest direct influence on NCD management. This may be due to having a good attitude; individuals are confident in practice towards the management of chronic disease [26]. Therefore, when individuals have a positive attitude, it helps to improve their management of NCDs. Additionally, findings revealed that community factors (neighborhood, local community, community organization, and healthcare services) influence NCD management. It indicated that community factors effectively influence NCD management. One probable explanation is that the approach of health promotion programs applied in the community has increased their awareness of NCD. This result was consistent with previous studies, which have reported that community-based programs, such as Komuniti Sihat Perkasa Negara (KOSPEN), help address NCDs and prevent and control NCD risk factors [27]. Another study discovered that community engagement involves partnerships with local health organizations, such as community outreach programs and hosting health awareness events, which contribute to the overall well-being of the communities [28]. Therefore, the involvement of community health workers as part of the healthcare team is one of the effective efforts of the community program in increasing access to treatment and healthcare. This indicated that the community approach initiative helps ongoing healthcare by preventing and controlling NCD risk within the community [29]. Another possible reason is that Putrajaya was designed with a neighborhood concept in mind, adopting a garden city concept that provides residents with neighborhood parks, including green

spaces, gardens, and lakes, promoting physical activity (e.g., walking, jogging, and cycling). A previous study noted that open spaces in Putrajaya significantly contribute to health promotion [30]. A similar finding was also noted, highlighting that the presence of green urban spaces promotes healthy communities and healthy lifestyles [31]. Thus, it encourages a healthier lifestyle through community engagement in outdoor activities, fostering community bonds in shared recreational spaces. Furthermore, since the respondents live in an urban area, the majority of them have easy access to healthcare services. Urban areas make it easier for people to access healthcare services due to better public transportation options and a variety of clinic and hospital services, which influence the management of NCDs. In fact, individuals from relatively low-income earners prefer to choose services from their community, where they need only pay MYR 1 for a general outpatient consultation and MYR 5 for a specialist consultation [32]. This is consistent with the report by NHMS 2019, which revealed that 8.1% of B40 households in Malaysia received healthcare services as outpatients in the two weeks prior to consulting healthcare professionals [2]. Moreover, the government's role at the primary care level in NCD management has seen an increase in the roles of doctors, nurses, and medical assistants, where follow-up care is provided at many primary health clinics [33]. This is supported by [34], who mentioned that management in the primary care system provides services to the community for effective disease management and patient empowerment. This subsequently leads to better health status among NCD patients as well as the general community. Furthermore, healthcare services help prevent and treat health problems for the public, which in turn influences the population's overall health status. Therefore, community factors such as access to healthcare facilities, availability of recreational spaces, and community health programs had an effect on NCD management among support staff.

This study also revealed that societal factors (health policy of healthcare financing, and mass media) were identified as significant factors that significantly influenced NCD management. This could be a health policy in healthcare financing by the government, helping to protect them from NCDs. This aligns with the findings of the NHMS (2009-2023), which reported that government spending on health, particularly for low-income groups, reflects a strong public role in health financing for these groups. For example, PeKa B40 aims to improve access to healthcare services for low-income groups [35]. In addition, heavily subsidized by the government, providing affordable and accessible care to all citizens, especially low-income groups. Therefore, government healthcare financing plays a significant role in protecting populations, especially low-income groups. On the other hand, mass media also play a vital role in influencing NCD management. The existence of mass media provides numerous benefits to the public, making it easier for them to obtain, share, and exchange information, which in turn influences healthy behavior. The findings are consistent with prior research indicating that social media influences preventive behavior by increasing awareness and facilitating information exchange [36]. Another study emphasized that the use of mass media influences health behavior [37]. This implies that NCD management can be improved by enhancing attitude, community, and societal factors.

The current study revealed that attitude mediates the relationship between intrapersonal, community, and societal factors on NCD management. This study found that community and societal factors were partially mediated by attitude. The partial mediating role of attitude was due to community and societal factors having direct and indirect relationships with NCD management through attitudes as mediators. Additionally, attitude fully mediates the relationship between the intrapersonal factor of knowledge and NCD management. This indicated that attitude helps

regulate the relationship between intrapersonal, community, and societal factors in NCD management. In other words, improvements in intrapersonal, community, and societal factors encouraged individuals to adopt better attitudes, which further enhanced NCD management. Furthermore, by increasing an individual's level of awareness, their building practices toward the prevention of NCD can be improved. The findings of this study were consistent with previous studies, which have proven attitude to be a mediator in controlling the relationship between exogenous variables and endogenous variables [38-40]. Hence, attitude serves as a mediator, influencing interpersonal, community, and societal factors in NCD management. Taken together, relevant stakeholders might better consider mediated relationships in a tailored intervention towards achieving NCD management.

Conclusion

In summary, attitude, community, and societal factors have direct effects on NCD management based on the results of the SEM analysis. The mediation test revealed that attitude acts as a partial mediator in the relationship between community and societal factors towards NCD management. Meanwhile, attitude serves as a full mediator in the relationship between intrapersonal factors and NCD management.

To the best of our knowledge, this is the first study to investigate the mediating effects of attitude on the relationship between socio-ecological factors and NCD management. This study presents a new approach by integrating socio-ecological factors through attitudes as a mediator in NCD management. The research also provides new insights for relevant stakeholders, including policymakers, healthcare

providers, and programme managers, by offering a better understanding of these influences and the socio-ecological approach to designing holistic interventions for the future. However, the study has limitations, such as the use of self-report questionnaires, which may have given false or incomplete responses. Additionally, the cross-sectional design makes it impossible to establish causality, which longitudinal studies could help determine. In addition, this study only utilized socio-ecological factors (e.g., intrapersonal, interpersonal, organizational, community, and societal). The assessment of other factors, such as health literacy and self-efficacy, should be considered in this study, as they can contribute to a deeper understanding of NCD management. Lastly, future studies can also be conducted on larger samples by utilizing SEM analysis to confirm the good results and generalizability.

References

1. Institute of Public Health, Technical Report National Health and Morbidity Survey (NMHS) 2023. Non-Communicable Disease and Healthcare Demand, Malaysia, 2024.

2. Institute of Public Health, National Health and Morbidity Survey 2019 (NHMS 2019). Non-communicable diseases, healthcare demand, and health literacy. Kuala Lumpur: Ministry of Health Malaysia, 2019.

3. Institute of Public Health, National Health and Morbidity Survey 2015 (NHMS 2015). Vol. II: Non-Communicable Diseases, Risk Factors & Other Health Problems. Kuala Lumpur: Ministry of Health Malaysia, 2015.

4. Budreviciute A, Damiati S, Sabir DK, Onder K, Schuller-Goetzburg P, Plakys G, , et al. Management and prevention strategies for non-communicable diseases (NCDs) and their risk factors. Front. public health. 2020; 8:574111. https://doi: 10.3389/fpubh.2020.574111.

5. Stringhini S, Carmeli C, Jokela M, Avendaño M, Muennig P, Guida F,et al., Socioeconomic status and the 25× 25 risk factors as determinants of premature mortality: a multicohort study and meta-analysis of 1· 7 million men and women. Lancet. 2017; 389 (10075):1229–1237. https://doi.org/10.1016/S0140-6736(16)32380-7.

6. Isamail N, Said R, Ismail NW, Haron SA. Non-Communicable Diseases Impact Low-Income Households in Malaysia. Malaysian Journal of Medical Sciences. 2024;31(1): 124. https://doi: 10.21315/mjms2024.31.1.11.

7. PhillipsCM, Chen LW, Heude B, Bernard JY, Harvey NC, Duijts L, et al. Dietary inflammatory index and non-communicable disease risk: a narrative review. Nutrients. 2019;11(8): 1873. https://doi: 10.3390/nu11081873.

8. Lee IM, Shiroma EJ, Lobelo F, Puska P, Blair SN, Katzmarzyk PT. Effect of physical inactivity on major non-communicable diseases worldwide: an analysis of burden of disease and life expectancy. Lancet. 2012;380 (9838): 219 –229. https://doi: 10.1016/S0140-6736(12)61031-9.

9. Lorber M, Reljić NM, Kegl B, Fekonja Z, Štiglic G, Davey A, et al. Person-Centred Care: A Support Strategy for Managing Non-Communicable Diseases. Healthcare. (2024);12(5): 526. https://doi.org/10.3390/healthcare12050526.

10. Nguyen KH, Fields JD, Cemballi AG, Desai R, Gopalan A, Cruz T, et al. The role of community-based organizations in improving chronic care for safety-net populations. Journal of the

American Board of Family Medicine. 2021;34 (4):698-708, https://doi: 10.3122/jabfm.2021.04.200591.

11. McLeroy KR, Bibeau D, Steckler A, Glanz K. An Ecological Perspective on Health Promotion Programs. Health Educ. Q. 1988; 315(4):51–377. https://doi: 10.1177/109019818801500401.
12. Baron SL, Beard S, Davis LK, Delp L, Forst L, Kidd-Taylor A,, et al. Promoting Integrated Approaches to Reducing Health Inequities Among Low- Income Workers: Applying a Social Ecological Framework. Am. J. Ind. Med. 2014;57: 539–556. https://doi: 10.1002/ajim.22174.
13. Hami R, Shahabudin SA, Shariff NM, Mohd Mujar NM, Isa S, Roslan H, et al. Factors Contributing to Non-Communicable Diseases in a Selected Low Socio-Economic Status Community in Kedah, Malaysia. Malaysian Journal of Medicine & Health Sciences. 2021;17(1): 117–123.
14. Harris H, Ooi YB, Lee JS, Matanjun P. Non-communicable diseases among low income adults in rural coastal communities in Eastern Sabah, Malaysia. BMC Public Health. 2019;19: 1–13. https://doi.org/10.1186/s12889-019-6854-6.
15. Fiidow OA, Huda BZ, Salmiah MS. Salmiah, Socio-demographic factors of hypertension among non-academic staff in Universiti Putra Malaysia. International Journal of Public Health and Clinical Sciences. 2016;3(4):158 –168.
16. SuTT, Amiri M, Mohd Hairi F, Thangiah N, Bulgiba A, Majid HA. Prediction of Cardiovascular Disease Risk among Low-Income Urban Dwellers in Metropolitan Kuala Lumpur, Malaysia. BioMed Res Int. 2015;2015(1): 516984. https:// doi: 10.1155/2015/516984.
17. Puteh SE, Kadir HA.. Factors associated with health status and living needs impact on b40 women's quality of life in Malaysia: an analysis based on b40 rural and urban poor areas.

Malaysian J. Public Health Med. 2022; 22(2):176–186. https://doi.org/10.37268/mjphm/vol.22/no.2/art.1717.

18. Shahar S, Lau H, Puteh SE, Amara S, Razak NA. Health, access and nutritional issues among low-income population in Malaysia: Introductory note. BMC Public Health. 2019;19: 1–5. https://doi.org/10.1186/s12889-019-6852-8.

19. Wan PutehSE, Siwar C, Zaidi MA, Abdul Kadir H. Health related quality of life (HRQOL) among low socioeconomic population in Malaysia. BMC Public Health. 2019;19: 1–8. https://doi.org/10.1186/s12889- 019-6853-7.

20. ÇabukS, Tanrikulu C, Gelibolu L. Understanding organic food consumption: attitude as a mediator. International Journal of Consumer Studies. 2014;38: 337–345. https://doi.org/10.1111/ijcs.12094.

21. Soper DS, A-Priori Sample Size Calculator for Structural Equation Models [Software]. http://wwwdanielsopercom/statcalc, 2020.

22. Kline RB. Principles and practice of Structural Equation Modelling (3rd ed). New York: The Guilford Press. 2010.

23. Ithnin M, Nor NA, Nordin NJ, Effendy NM, Sahar MA, Abdullah KH,, et al. Knowledge, attitude, and practice on Non- Communicable Diseases (NCDs) among the adult population in the urban area of Negeri Sembilan, Malaysia. International Journal of Research in Pharmaceutical Sciences. 2018; 9 (2Suppl): 88–94. https://doi.org/10.1080/14635240.2020.1749526.

24. Glasgow RE, Strycker LA, Toobert DJ, Eakin E. A Social-Ecologic Approach to Assessing Support for Disease Self- Management: The Chronic Illness Resources Surveys. J. Behav. Med. 2000;23(6): 559-583. https:// doi:.10.1023/a:1005507603901.

25. Hair F, Black WC, Anderson RE. Multivariate Data Analysis. (7th Ed). New York: Pearson. 2010.

26. Ithnin M, Nor NA, Juliana N, Effendy NM, Sahar MA, Abdullah KH, et al. Knowledge, Attitude and Practices Towards Lifestyle Related Non-Communicable Diseases (NCDs): A Cross Sectional Study among Indigenous Orang Asli Adults in Negeri Sembilan, Malaysia. IIUM Medical Journal Malaysia. 2020;19 (2):75–82. https://doi.org/10.31436/imjm.v19i2.1569.

27. Lim KH, M Fadhli Y, Omar M, Rosnah R, M. Nazaruddin B, Sumarni MG, et al. Evulation of Effectiveness of Implementation of "Komuniti Sihat Perkasa Negara (KOSPEN) Programme in Malaysia- Phase 1. Kuala Lumpur: Ministry of Health Malaysia, 2015.

28. Kabuya KE. Influence of Workplace Health on Awareness of Non-Communicable Diseases (NCDs) and Its Cascading Effects on the Well-being of Families and Communities. International Journal of Sciences: Basic and Applied Research. 2024. 73 (1):43–58.

29. Kataria I, Ngongo C, Lim SC, Kocher E, Kowal P, Chandran A, et al. Development and evaluation of a digital, community-based intervention to reduce noncommunicable disease risk in a low-resource urban setting in Malaysia: a research protocol. Implementation science communications. 2020;1: 1–9. https://doi.org/10.1186/s43058-020-00080-y.

30. Danjaji AS, Danladi M, Adamu A, Danladi HM. Urban Green Spaces as Promoters of Healthy Living: Evidence from Putrajaya, Malaysia. Research Square. 2021: 1 –17. https://doi.org/10.21203/rs.3.rs-1166766/v1.

31. Ayob Z, A.R. Amir AR. Developing Urban Walkability Measures Grounded in Green Urbanism Principles Using The Delphi Survey Study. Planning Malaysia. 2024; 22:307–320.

32. Aizuddin AN, Al Junid SM. Willingness to pay for outpatient services user fees: Malaysian community perspective. Jurnal Sains Kesihatan Malaysia. 2018; 16(1):145-153.

33. Mustapha FI, Omar ZA, Mihat O, Md Noh K, Hassan N, Abu Bakar R, et al. Addressing non-communicable diseases in Malaysia: an integrative process of systems and community. BMC Public Health. 2014;14: 1–6. htttps://doi:doi: 10.1186/1471-2458-14-S2-S4.

34. Fadzilah A, Azima R, Khalsom S, Rosliza AM. Governance framework in non-communicable disease (NCD) control and prevention programme at primary care level in Malaysia. International Journal of Public Health and Clinical Sciences. (2020); 7(3):167-185.

35. Yunus SZ, Puteh SE, Ali AM, Daud F., S.E.W. Puteh, A.M. Ali, F. Daud, The Covid impact to public healthcare utilization among urban low-income subsidized community in Klang Valley Malaysia. Health Serv. Res. Manag, Epidemiol. 2021;8: 1–9. https://doi: 10.1177/23333928211002407.

36. Nazir M, Hussain I, Tian J, Akram S, Mangenda Tshiaba S, Mushtaq S, et al. A multidimensional model of public health approaches against COVID-19. International Journal of Environmental Research and Public Health. 2020; 17(11): (3780. https://doi.org/10.3390/ijerph17113780.

37. Nabi RL, Huskey R, Nicholls SB, Keblusek L, Reed M. When audiences become advocates: Self-induced behavior change through health message posting in social media. Computers in Human Behavior. 2019;99: 260–267. https://doi.org/10.1016/j.chb.2019.05.030.

38. Zhang Y, Zhang B, Chen C, Feng X, Song S, Wang H. The mediation effect of attitude on the association between knowledge and self-management behaviors in Chinese patients with diabetes. International Journal of Public Health. 2023; 68: 1606022. https;//doi: 10.3389/ijph.2023.1606022.

39. Saengcha S, Pattanapongthorn J, Jermsittiparsert K. Influence of Beliefs on Attitudes and Intention of Adopting e-Health System among the Public Hospitals of Thailand. International Journal of Innovation, Creativity and Change. 2019; 8(8):238–255.

40. An HG, Ha JY, Kim SH. Mediating Effect of Attitude on the Relationship between Knowledge about Tuberculosis and Preventive Behavior Toward Tuberculosis of Foreign Students. International Journal of Bio-Science and Bio-Technology. 2016;8(2):181–196. https://dx.doi.org/10.14257/ijbsbt.2016.8.2.17.

Dear Editor,

I would like to express my gratitude to the reviewers for their helpful criticisms and comments, which have enabled us to improve the quality of this manuscript.

Please refer to the manuscript highlighted in blue for our responses to each of the reviewers' comments and concerns below.

No.	Reviewer Comment	Response
1.	The justification for selecting the government servant is unclear. Please also justify selecting only 8 ministries randomly rather than taking up representatives from each ministries to make up the sample size.	A cluster random sampling technique was employed in this study. Of 24 ministries, eight ministries were randomly selected to represent the ministries in Putrajaya. Subsequently, the respondents were selected using random sampling based on the criteria: permanent support staff aged between 18 and 60 years old and having NCDs. Meanwhile, exclusion criteria were contract or part-time staff and those who refused to participate.
2.	Typo in spelling	Sample Size
3.	Please add how the questionnaire survey were distributed.	A set of questionnaires was distributed to respondents who fulfilled the inclusion criteria via an online survey using a Google Form link.
4.	What was the modification made? Please justify the need for the modification.	The questionnaire was adapted from previous studies
5.	Typo in spelling	Section B assesses the respondents' knowledge of NCD.
6.	No?	I have reworded sentences in line with your suggestion. No
7.	Rephrase the description for the scores as it in grammartically incorrect to mix up active and passive sentences.	Knowledge levels were classified as poor (0-50%), moderate (51-75%), and good (76-100%).
8.	Briefly explain why this is needed for this study.	The Cronbach's Alpha for the dimensions of the questionnaire ranged from 0.70 to 0.90, demonstrating good reliability.
9.	Age percentage not included	The majority of the respondents were aged between 40 and 49 years old (67.5%).
10.	Rephrase this sentence on the sociodemographic analysis. Less than half is not an academic phrase of reporting.	The household income of the respondents ranges between RM3501-RM4000 (51.0%), and they received a higher educational level of at STPM/ Matriculation/ Diploma and SPM/Certificate (69.0%).
11.	Unclear statement, not having the diseases? Justify why this data is necessary to the study.	Thank you for your valuable suggestion. I acknowledge that the data is unnecessary and deleted from the manuscript.
12.	Readers would benefit on how SEM is generally used to achieve this study's	I have revised this section to explain SEM clearly.

	objectives. a brief idea on how and why SEM works. CFI, TLI and other values mentioned are can be made useful for readers who are not familiar with SEM.	Structural Equation Modeling (SEM) was employed to assess the hypotheses of the study. Using this method, hypotheses can explain relationships among multiple variables and the degree of their correlations with the data obtained simultaneously. Subsequently, the model fit was determined based on the Goodness-of-Fit (GOF) [25]. GOF is based on how well the data predicted by the model correspond to the data that were actually collected. The closer the values of these two matrices are to each other, the better the model is considered to fit. GOF indices should be reported include $\chi2$ value, relative ($\chi2$/df), absolute fit indices (i.e., Goodness-of-Fit Index (GFI), and Root Mean Square Error of Approximation (RMSEA), and incremental fit indices (i.e., Comparative Fit Index (CFI), Normed Fit Index (NFI), and Tucker-Lewis Index (TLI)). Based on the GOF indices, the model is considered fit when the values of the GFI and the Adjusted Goodness-of-Fit Index (AGFI) range between 0 and 1. However, values greater than 0.9 indicate a better and more meaningful model fit. Furthermore, the incremental fit indices such as the CFI, NFI, Incremental Fit Index (IFI), and TLI also suggest a good model fit when their values are approximately 0.9 or higher, with a value of 1 representing a perfect fit to the model. RMSEA is another important measure, where values greater than .1 indicate a poor model fit, while values ranging from .08 to .1 indicate a mediocre fit. Moreover, values ranging from .03 and .08 indicate a better fit model, whereas Chisq/df lower than the upper limit should be from 1.0 to 3.0, while the maximum acceptable limit is 5.0.<br>Hair et al. (2010) recommended that three to four indices, including one absolute index and one incremental index, meet the criteria, which provides adequate evidence of model fit [25].Based on SEM, the resulting structural model fits indicated the data fit the model with [ChiSq/df = 1.693, P = 0.000, CFI = 0.920, TLI = 0.912, RMSEA = 0.059]. The value of the relative chi-square ($\chi2$/df) was well below 5, indicating an acceptable fit between the proposed measurement model and the collected data. Other fit indices, namely CFI and TLI values, were above the minimum value of .90, and the RMSEA value was below the cut-off value

		of .08 (RMSEA = .050). Therefore, the analysis of the SEM indicated that the overall value of the fit index reached acceptable levels and confirmed that the structural model was a good fit.
13.	Again, briefly explain how does causal path analysis work.	As suggested by the reviewer, we have explained the structural model analysis was used to conduct path analysis and test the research hypotheses.
14.	Several other studies were used to support some of the findings here. How does your study differ and what's new worth highlighted here?	To the best of our knowledge, this is the first study to investigate the mediating effects of attitude on the relationship between socio-ecological factors and NCD management. This study presents a new approach by integrating socio-ecological factors through attitudes as a mediator in NCD management. The research also provides new insights for relevant stakeholders, including policymakers, healthcare providers, and programme managers, by offering a better understanding of these influences and the socio-ecological approach to designing holistic interventions for the future. However, the study has limitations, such as the use of self-report questionnaires, which may have given false or incomplete responses. Additionally, the cross-sectional design makes it impossible to establish causality, which longitudinal studies could help determine. In addition, this study only utilized socio-ecological factors (e.g., intrapersonal, interpersonal, organizational, community, and societal). The assessment of other factors, such as health literacy and self-efficacy, should be considered in this study, as they can contribute to a deeper understanding of NCD management. Lastly, future studies can also be conducted on larger samples by utilizing SEM analysis to confirm the good results and generalizability.
