# Supplementary material for: Determinants of non-communicable disease management among support staff in Putrajaya, Malaysia: The mediating role of attitude
Source: PLoS One. 2026 Apr 30;21(4):e0347528. doi: 10.1371/journal.pone.0347528 (PMC13132246; doi:10.1371/journal.pone.0347528)
Supplement: Attachment — Submitted filename: Response to Reviewers.docx [file pone.0347528.s004.docx]

Dear Editor,

I would like to express my gratitude to the reviewers for their helpful criticisms and comments, which have enabled us to improve the quality of this manuscript.

Please refer to the manuscript highlighted in blue for our responses to each of the reviewers’ comments and concerns below.

| **No.** | **Reviewer Comment** | **Response** |
| --- | --- | --- |
| 1. | The justification for selecting the government servant is unclear. Please also justify selecting only 8 ministries randomly rather than taking up representatives from each ministries to make up the sample size. | A cluster random sampling technique was employed in this study. Of 24 ministries, eight ministries were randomly selected to represent the ministries in Putrajaya. Subsequently, the respondents were selected using random sampling based on the criteria: permanent support staff aged between 18 and 60 years old and having NCDs. Meanwhile, exclusion criteria were contract or part-time staff and those who refused to participate. |
| 2. | Typo in spelling | Sample Size |
| 3. | Please add how the questionnaire survey were distributed. | A set of questionnaires was distributed to respondents who fulfilled the inclusion criteria via an online survey using a Google Form link. |
| 4. | What was the modification made? Please justify the need for the modification. | The questionnaire was adapted from previous studies |
| 5. | Typo in spelling | Section B assesses the respondents’ knowledge of NCD. |
| 6. | No? | I have reworded sentences in line with your suggestion. No |
| 7. | Rephrase the description for the scores as it in grammartically incorrect to mix up active and passive sentences. | Knowledge levels were classified as poor (0-50%), moderate (51-75%), and good (76-100%). |
| 8. | Briefly explain why this is needed for this study. | The Cronbach’s Alpha for the dimensions of the questionnaire ranged from 0.70 to 0.90, demonstrating good reliability. |
| 9. | Age percentage not included | The majority of the respondents were aged between 40 and 49 years old (67.5%). |
| 10. | Rephrase this sentence on the sociodemographic analysis. Less than half is not an academic phrase of reporting. | The household income of the respondents ranges between RM3501-RM4000 (51.0%), and they received a higher educational level of at STPM/ Matriculation/ Diploma and SPM/Certificate (69.0%). |
| 11. | Unclear statement, not having the diseases? Justify why this data is necessary to the study. | Thank you for your valuable suggestion. I acknowledge that the data is unnecessary and deleted from the manuscript. |
| 12. | Readers would benefit on how SEM is generally used to achieve this study's objectives. a brief idea on how and why SEM works. CFI, TLI and other values mentioned are can be made useful for readers who are not familiar with SEM. | I have revised this section to explain SEM clearly.  Structural Equation Modeling (SEM) was employed to assess the hypotheses of the study. Using this method, hypotheses can explain relationships among multiple variables and the degree of their correlations with the data obtained simultaneously. Subsequently, the model fit was determined based on the Goodness-of-Fit (GOF) [25]. GOF is based on how well the data predicted by the model correspond to the data that were actually collected. The closer the values of these two matrices are to each other, the better the model is considered to fit. GOF indices should be reported include χ2 value, relative (χ2/df), absolute fit indices (i.e., Goodness-of-Fit Index (GFI), and Root Mean Square Error of Approximation (RMSEA), and incremental fit indices (i.e., Comparative Fit Index (CFI), Normed Fit Index (NFI), and Tucker-Lewis Index (TLI)). Based on the GOF indices, the model is considered fit when the values of the GFI and the Adjusted Goodness-of-Fit Index (AGFI) range between 0 and 1. However, values greater than 0.9 indicate a better and more meaningful model fit. Furthermore, the incremental fit indices such as the CFI, NFI, Incremental Fit Index (IFI), and TLI also suggest a good model fit when their values are approximately 0.9 or higher, with a value of 1 representing a perfect fit to the model. RMSEA is another important measure, where values greater than .1 indicate a poor model fit, while values ranging from .08 to .1 indicate a mediocre fit. Moreover, values ranging from .03 and .08 indicate a better fit model, whereas Chisq/df lower than the upper limit should be from 1.0 to 3.0, while the maximum acceptable limit is 5.0.  Hair et al. (2010) recommended that three to four indices, including one absolute index and one incremental index, meet the criteria, which provides adequate evidence of model fit [25].Based on SEM, the resulting structural model fits indicated the data fit the model with [ChiSq/df = 1.693, P = 0.000, CFI = 0.920, TLI = 0.912, RMSEA = 0.059]. The value of the relative chi-square (χ2/df) was well below 5, indicating an acceptable fit between the proposed measurement model and the collected data. Other fit indices, namely CFI and TLI values, were above the minimum value of .90, and the RMSEA value was below the cut-off value of .08 (RMSEA = .050). Therefore, the analysis of the SEM indicated that the overall value of the fit index reached acceptable levels and confirmed that the structural model was a good fit. |
| 13. | Again, briefly explain how does causal path analysis work. | As suggested by the reviewer, we have explained the structural model analysis was used to conduct path analysis and test the research hypotheses. |
| 14. | Several other studies were used to support some of the findings here. How does your study differ and what's new worth highlighted here? | To the best of our knowledge, this is the first study to investigate the mediating effects of attitude on the relationship between socio-ecological factors and NCD management. This study presents a new approach by integrating socio-ecological factors through attitudes as a mediator in NCD management. The research also provides new insights for relevant stakeholders, including policymakers, healthcare providers, and programme managers, by offering a better understanding of these influences and the socio-ecological approach to designing holistic interventions for the future. However, the study has limitations, such as the use of self-report questionnaires, which may have given false or incomplete responses. Additionally, the cross-sectional design makes it impossible to establish causality, which longitudinal studies could help determine. In addition, this study only utilized socio-ecological factors (e.g., intrapersonal, interpersonal, organizational, community, and societal). The assessment of other factors, such as health literacy and self-efficacy, should be considered in this study, as they can contribute to a deeper understanding of NCD management. Lastly, future studies can also be conducted on larger samples by utilizing SEM analysis to confirm the good results and generalizability. |
