# Supplementary material for: Determinants of non-communicable disease management among support staff in Putrajaya, Malaysia: The mediating role of attitude
Source: PLoS One. 2026 Apr 30;21(4):e0347528. doi: 10.1371/journal.pone.0347528 (PMC13132246; doi:10.1371/journal.pone.0347528)
Supplement: Attachment — Submitted filename: Response_to_Reviewers_auresp_2.docx [file pone.0347528.s006.docx]

| **No.** | **Comments** | **Response** |
| --- | --- | --- |
| 1. | Please revise this statement to reflect how attitude mediates the effect of SEM on NCD management. | Thank you for the comment. The manuscript has been revised to clearly reflect that attitudes function as a mediating mechanism in the relationship between intrapersonal factors and NCD management. |
| 2. | Main issue of the mediating effect of attitude did not transpire in the main suggestion of the study. | Thank you for these valuable comments. The implication statement has been rephrased to highlight the importance of mediated relationships that can be translated into effective NCD management strategies. |
| 3. | Hanging sentence. | The sentence has been revised to address the grammatical issue by completing the clause introduced by “Despite” and clearly linking it to the main idea. This revision improves clarity and ensures the sentence is grammatically complete. |
| 4. | Comma as separator. | Thank you for the comment. The sentence has standardized the use of commas in the list. |
| 5. | McLeroy et al. indicated that individual/intrapersonal includes personal characteristics such as knowledge, attitudes, beliefs, and behaviors that influence health decisions. Please justify why there is a need to have a separate Attitude construct in the theoretical model? | Thank you for this important comment. We acknowledge that within the Socio-Ecological Model, attitude is commonly conceptualized as part of the intrapersonal level. However, in this study, attitude is modeled as a distinct construct to examine its specific mediating role between intrapersonal factor (knowledge) and NCD management. This approach is theoretically grounded in health behavior models such as the Knowledge–Attitude–Practice (KAP) model, which conceptualizes attitude as a key psychological mechanism linking cognitive and contextual influences to behavioral outcomes. The manuscript has been revised to clarify this theoretical justification and to explicitly explain the rationale for modeling attitude as a separate mediator. Therefore, modeling attitude as a separate mediator offers valuable insights and provides a clearer explanation of how socio-ecological factors (intrapersonal) influenced NCD management. |
| 6. | Please provide reference for this statement. | Thank you for the comment. Appropriate references have now been added to support the statements in the manuscript. |
| 7. | Is attitude measured by SEM model? If it is not, then this sentence might not be suitable. | Thank you for the comment. In this study, attitude is explicitly measured and modeled within the Structural Equation Modeling (SEM) framework as a latent construct and tested as a mediator between intrapersonal factors (knowledge) and NCD management. The manuscript has been revised to clearly state this methodological approach and to strengthen the justification regarding the limited use of SEM in local studies examining the mediating role of attitude within a socio-ecological framework. Therefore, attitude is not merely a conceptual element but is empirically measured and tested within the SEM model. |
| 8. | I suggest author to include theoretical framework of the relationship and consequently explain why need to examine mediation effect. | The theoretical framework of this study is presented in Figure 1. |
| 9. | Suggest to provide a reference for these facts. | Thank you for the comment. Relevant references have been added to support these statements in the manuscript |
|  | Technically, if a cluster random sampling technique is employed, all samples in the cluster should be selected but this is not the case in this study. Therefore, it is inappropriate to state that this study used cluster random sampling. Please refer to a statistician to determine the actual sampling technique that has been used by this study. | Thank you for the reviewer’s insightful comment. We acknowledge that the term “cluster random sampling” was not used precisely in the original manuscript. Therefore, the sampling strategy has been clarified and revised to reflect a two-stage cluster sampling approach, where ministries were first selected as clusters, followed by simple random sampling of eligible individuals within each selected cluster using a computer-generated random number procedure. The manuscript has been revised accordingly. |
| 10. | Please also describe how random selection of participants were carried out. Is it using random number generator and selecting respondents from a listed names of support staff in the ministries? | Thank you for the comment. The manuscript has been revised to clearly describe the random selection procedure. Specifically, a list of eligible support staff was obtained from the Human Resources Division of the selected ministries. Each individual was assigned a unique identification number, and participants were randomly selected using a computer-generated random number procedure (simple random sampling). This clarification has been added to the Methods section under the sampling procedure |
| 11. | This paper has used SEM for 2 different contexts. This might confuse the readers. Please differentiate the abbreviations. | Thank you for highlighting this issue. We agree that using the abbreviation “SEM” for two different concepts may confuse. In this study, the Socio-Ecological Model (McLeroy, 1988) is used as the theoretical framework, whereas Structural Equation Modeling (SEM) refers to the statistical analysis technique employed to test the hypothesized relationships.  To avoid confusion, the abbreviation “SEM” is now used exclusively to denote Structural Equation Modeling throughout the manuscript, while the Socio-Ecological Model is referred to in full without abbreviation. |
| 12. | Please indicate the source of determining these values. | Thank you for the reviewer’s comment. These values are directly derived from the structure of the measurement model used in this study (Glasgow et al., 2000; Ithin et al., 2018). |
| 13. | Reporting Cronbach's Alpha only is not sufficient. The author also need to report construct validity results. prior to present SEM analysis results. | We thank the reviewer for the valuable comment. In response, we have added construct validity results prior to presenting the SEM analysis.The Average Variance Extracted (AVE) values ranged from 0.463 to 0.730, and Composite Reliability (CR) values ranged from 0.778 to 0.955, indicating adequate convergent validity. Additionally, the square roots of AVE values for all sub-constructs were higher than the inter-construct correlations, confirming that discriminant validity was achieved. These results demonstrate that the measurement model meets the requirements for SEM analysis. |
| 14. | SEM for Socio-Ecological Model OR Structural Equation Model? | Thank you for the reviewer’s comment. We would like to clarify that in this study, SEM refers to Structural Equation Modeling used to examine the interrelationships among the constructs in the hypothesized model. |
| 15. | These sentences should be a continuation from previous one. Please make amendment. | We thank the reviewer for the comment. We have amended the sentences so that they now continue smoothly from the previous one. |
|  | What are the hypotheses of the study? | We thank the reviewer for the comment. The study hypotheses were amended. Specifically, the hypotheses aim to determine factors influencing NCD management and the mediating role of attitude in these relationships. |
| 16. | This sentences are not appropriate to be included in the result section. | We thank the reviewer for the comment. We agree that the sentences were not appropriate for the results section. Accordingly, these sentences have been removed from the results |
| 17. | The results has a negative coefficient. | Thank you for the comment.We would like to clarify that the standardized coefficient for community factors is indeed negative (β = -0.259, p < 0.001), |
| 18. | This paper lack ground basis why there is a need to have additional attitude construct with SEM on the NCD management. | We thank the reviewer for the comment. The inclusion of the attitude construct in the SEM model is theoretically grounded in the Knowledge–Attitude–Practice (KAP) framework, which posits that knowledge and socio-ecological factors shape attitudes, which in turn influence health-related behaviors such as NCD management. In our model, attitude was included to assess both its direct effect on NCD management and its mediating role. The SEM results indicated that attitude has a significant direct effect on NCD management, while also partially mediating the relationship between community and societal factors, and fully mediating the relationship between intrapersonal factors and NCD management. This approach allows a more comprehensive understanding of how socio-ecological factors translate into NCD management behaviors. |
